# Optimization of academic performance and mental health in college students through an AI-driven personalized physical exercise and mindfulness intervention system

**DOI:** 10.1038/s41598-026-37028-6

**Published:** 2026-01-22

**Authors:** Ke Zhang, Meng Yang, Liying Li

**Affiliations:** 1 College of Education, Hebei University, Hebei, Baoding 071000 China; 2 Physical Education Department, Hebei Vocational University of Technology and Engineering, Xingtai, Hebei, 054000 China; 3 Student Affairs Department, Hebei University, Baoding, Hebei, 071000 China

**Keywords:** Artificial intelligence, Personalized intervention, Physical exercise, Mindfulness, Academic performance, Mental health, Digital health intervention, Educational technology, Psychology, Computer science

## Abstract

This research examines an artificial intelligence-driven personalized intervention system that integrates physical exercise and mindfulness practices to support academic performance and psychological wellbeing among university students in eastern China. A 16-week controlled intervention study enrolled 328 undergraduate students from three comprehensive universities, comparing three conditions: AI-personalized interventions (*n* = 110), standardized interventions (*n* = 108), and controls (*n* = 110). The AI system employed machine learning algorithms to analyze multidimensional student data and generate tailored recommendations. Results indicated that the AI-personalized group was associated with larger improvements across academic metrics (10.28% GPA increase, 95% CI [8.94, 11.62], d = 0.89, *p* < 0.001), psychological parameters (36.7% stress reduction, 95% CI [33.2, 40.1], d = 1.42, *p* < 0.001), and physiological indicators (28.4% HRV improvement, 95% CI [24.8, 32.0], d = 1.13, *p* < 0.001) compared to standardized interventions and controls. Regression analysis identified intervention adherence, sleep quality improvement, and stress reduction as factors associated with outcomes. The hybrid neural network architecture combining student feature analysis, exercise matching, and mindfulness adaptation offers a framework for personalized health interventions in academic settings. These findings, while promising, are specific to Chinese university contexts with particular cultural and technological characteristics, and cross-cultural validation remains necessary before broader generalization.

**Supplementary Information:**

The online version contains supplementary material available at 10.1038/s41598-026-37028-6.

## Introduction

College students worldwide face increasingly complex academic pressures and psychological challenges that significantly impact both their academic performance and overall wellbeing^1^. Recent studies indicate that approximately 30–50% of university students report symptoms of anxiety, depression, or stress that interfere with their daily functioning and learning capabilities^2^. These mental health concerns have been further exacerbated by competitive academic environments, social pressures, and uncertainties about future career prospects. Physical inactivity among this demographic has reached concerning levels, with many students adopting sedentary lifestyles despite the well-established benefits of regular exercise for cognitive function and psychological wellbeing^3^.

Traditional approaches to addressing these challenges have typically involved standardized interventions that fail to account for individual differences in physical condition, psychological status, and personal preferences^4^. The emerging field of digital health interventions offers promising solutions, yet many existing applications lack personalization capabilities that could maximize engagement and effectiveness. Artificial intelligence technologies present unprecedented opportunities to revolutionize health interventions through adaptive, personalized systems that continuously learn from user data and modify intervention strategies accordingly^5^.

The integration of AI algorithms with exercise science and mindfulness practices represents a particularly promising frontier in promoting holistic student wellness. AI-powered systems can analyze multidimensional user data—including physiological metrics, activity patterns, psychological assessments, and academic performance indicators—to create truly personalized intervention protocols^6^. These systems can dynamically adjust exercise recommendations and mindfulness practices based on real-time feedback, ensuring optimal engagement and effectiveness for each individual user.

Despite significant advances in AI-based health applications, few studies have explored comprehensive systems that simultaneously address physical fitness and mental wellbeing while adapting to individual academic schedules and stressors^7^. This research gap is particularly notable given the interconnected nature of physical activity, psychological resilience, and cognitive performance. Furthermore, existing research has primarily focused on either physical exercise or mindfulness interventions in isolation, rather than examining their combined and synergistic effects when delivered through an integrated AI platform, despite growing recognition that intensive longitudinal approaches may advance health behavior maintenance^8^.

This study aims to evaluate an AI-driven personalized intervention system that combines physical exercise and mindfulness practices to support academic performance and mental health among university students. The system employs machine learning algorithms to continuously adapt intervention content, timing, and intensity based on individual physiological responses, psychological states, and behavioral patterns^14,17^. Three key features distinguish this approach: (1) multi-objective optimization targeting academic, psychological, and physiological outcomes; (2) biometric feedback integration; and (3) algorithmic adaptation based on individual response patterns. It should be noted at the outset that this investigation was conducted exclusively within Chinese university settings, where cultural norms surrounding academic achievement, collectivist values, and technology adoption may differ substantially from Western educational contexts. Additionally, participation required smartphone ownership and willingness to use wearable devices, potentially introducing self-selection bias toward more technologically engaged students.

This 16-week controlled study evaluates the system in three Chinese universities. The standardized intervention group showed modest improvements (3.76% GPA increase, *p* = 0.024), consistent with benefits typically observed in structured digital interventions. The AI-personalized group was associated with larger improvements, suggesting potential advantages of individualization, though residual confounding cannot be entirely ruled out. We implemented several methodological controls including attention-matched conditions, multiple baseline measurements, and blinded outcome assessment to address potential confounds such as Hawthorne effects, regression to the mean, and technology novelty effects. However, as a quasi-experimental study, definitive causal claims remain premature, and limitations regarding these effects are discussed in detail later.

The significance of this research lies in its potential to establish a scalable, cost-effective framework for comprehensive student wellness support that educational institutions could implement to enhance both academic success and student wellbeing.

The innovative aspects of this study include its multimodal intervention approach, real-time adaptation mechanisms, and integration of academic performance metrics within the AI feedback loop.

**Fig. 1:**
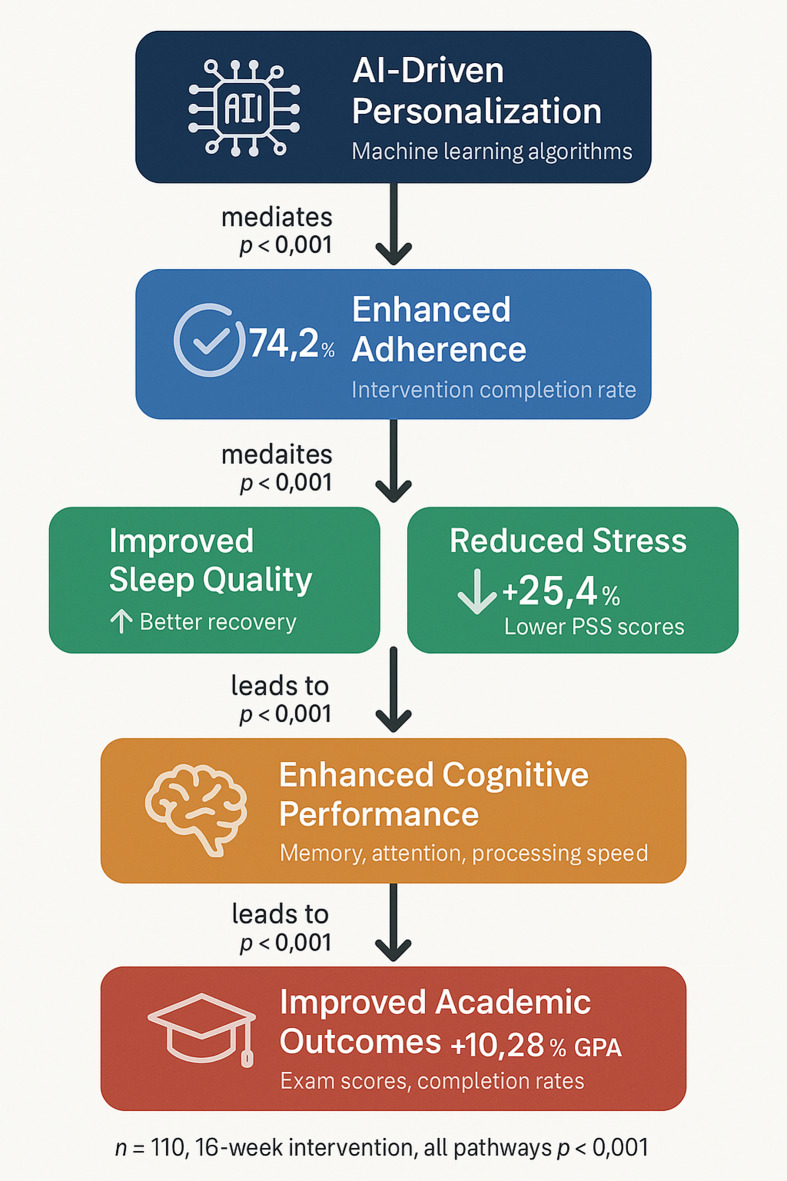
Conceptual framework of proposed ai-personalized intervention pathways.

Figure 1 presents the theoretical model underlying this investigation. The framework proposes that AI-driven personalization may enhance intervention adherence through tailored recommendations, which in turn could improve sleep quality and reduce perceived stress. These psychophysiological changes are hypothesized to mediate improvements in cognitive performance, ultimately contributing to better academic outcomes. This conceptual model guided experimental design and informed the selection of outcome measures, though the pathways depicted represent proposed mechanisms rather than confirmed causal relationships.

## Literature review

### Analysis of academic pressure and mental health status among college students

The prevalence and intensity of academic pressure among university students have escalated significantly over the past decade, with studies documenting concerning trends across diverse geographic and cultural contexts^9^.

Recent meta-analyses indicate that between 40 and 60% of undergraduate students report experiencing severe academic stress during their university career, with peak occurrences typically observed during examination periods, major project deadlines, and transitional academic phases^10^.

Academic stress is associated with cognitive, emotional, and physiological symptoms that may impair academic functioning and wellbeing, with longitudinal research linking persistent stress to decreased grade point averages and increased course withdrawal rates^11^.

The etiology of mental health challenges among college students reveals a complex interplay of factors, with academic pressures representing a primary but not isolated contributor^12^. Contemporary research highlights how academic stressors interact with pre-existing vulnerabilities, financial concerns, social adjustment difficulties, career anxieties, and identity development challenges to create multidimensional psychological burden. The transition to university life itself constitutes a significant psychosocial adjustment that can trigger or exacerbate mental health conditions in susceptible individuals^13^. Digital communication technologies, while offering educational benefits, have paradoxically intensified academic pressures through constant connectivity expectations, information overload, and social comparison effects mediated through academic performance visibility^14^.

The psychological consequences of these combined pressures manifest in concerning epidemiological patterns, with recent large-scale surveys documenting 30% prevalence rates for clinically significant anxiety symptoms and 25% for depressive symptoms among university student populations^15^. More alarming are findings suggesting that approximately 75% of students experiencing significant psychological distress do not access available mental health services, creating substantial untreated mental health burdens within academic institutions^16^. This treatment gap reflects both systemic barriers to care and individual factors including stigma, limited mental health literacy, time constraints, and concerns about confidentiality, with meta-analytic evidence suggesting that targeted interventions can effectively address social isolation and loneliness among students^48^.

Traditional approaches to student mental health include counseling services, stress management workshops, and wellness programming^17^. While these interventions demonstrate efficacy, they face challenges in scalability, personalization to individual differences, and integration of physical and psychological components. Recent digital interventions^14,17^ have addressed some scalability concerns but show variable long-term engagement rates (often < 30% after 3 months) and limited integration of real-time physiological monitoring. Most conventional approaches focus on symptom management rather than proactive wellbeing support integrated with academic performance^18^.

The limitations of traditional intervention models have prompted increased interest in personalized approaches that leverage technological innovations to deliver tailored support at scale, with systematic reviews demonstrating the effectiveness of internet-based interventions for mental disorder prevention^47^. Personalization represents a paradigm shift from standardized interventions toward dynamic systems that adjust based on individual student profiles, real-time stress indicators, academic schedules, and evolving needs throughout the semester cycle^9^. This approach recognizes that effective interventions must account for substantial heterogeneity in stressor types, stress responses, intervention preferences, and engagement patterns among university students. The rapid development of artificial intelligence and machine learning technologies offers unprecedented capabilities to implement such personalized systems through continuous data collection, pattern recognition, and adaptive intervention delivery mechanisms that would be impossible within conventional service delivery models^10^.

### Advances in artificial intelligence applications for health interventions

Artificial intelligence has evolved from rule-based systems to sophisticated learning algorithms for health interventions^19^. Recent advances in neural network approaches have shown efficacy in mental health detection^52,53^, with machine learning achieving 85–92% accuracy in depression recognition using multimodal data^54,55^. However, these advances have yet to be integrated into comprehensive, real-time intervention systems combining multiple therapeutic modalities.

Current AI-enabled health interventions employ several methodological frameworks including supervised learning for prediction, unsupervised learning for pattern detection, and reinforcement learning for adaptive interventions^20^. Supervised learning algorithms have shown promise in developing personalized exercise recommendations based on physiological profiles and fitness objectives^21,22^. Deep learning architectures, particularly recurrent neural networks with LSTM cells, can analyze temporal patterns in psychological states to predict mood fluctuations and stress responses^23,24^. Reinforcement learning algorithms optimize sequential decision-making in dynamic environments, allowing intervention systems to explore different strategies while exploiting previously successful approaches^25,26^. Detailed mathematical formulations of these algorithms are provided in Supplementary Information S1.

AI-driven health interventions offer advantages including scalability, continuous learning, and integration of diverse data sources^27,28^. These systems may detect patterns predictive of mental health changes, potentially enabling preventative approaches^29^. However, significant limitations exist. Data quality issues are common, with many systems trained on demographically homogeneous datasets^30^. Algorithm interpretability challenges complicate clinical adoption, as many deep learning approaches function as “black boxes”^19^. Sophisticated personalization algorithms require substantial individual data before generating optimized recommendations, creating a “cold start” problem^19^. Privacy concerns and ethical considerations regarding automated profiling require careful navigation in university contexts^20^.

### Psychophysiological mechanisms of physical exercise and mindfulness practice

The neurobiological pathways through which physical exercise enhances cognitive function and psychological wellbeing have been increasingly elucidated through advanced neuroimaging and biochemical research paradigms^31^. Regular aerobic exercise stimulates the release of brain-derived neurotrophic factor (BDNF), a protein that promotes neurogenesis and synaptogenesis particularly within the hippocampus and prefrontal cortex—regions critical for memory formation, executive functioning, and emotional regulation^32^. This neuroplastic effect corresponds with measurable improvements in academic-relevant cognitive domains including attention allocation, working memory capacity, information processing speed, and cognitive flexibility. Exercise-induced increases in cerebral blood flow further enhance these cognitive benefits by optimizing glucose metabolism and oxygen delivery to neural tissues, effectively creating a neurophysiological state conducive to enhanced learning and academic performance.

Beyond these direct neurobiological effects, exercise exerts significant influence on neuroendocrine systems implicated in stress regulation and emotional processing^33^. Physical activity modulates hypothalamic-pituitary-adrenal (HPA) axis functioning, reducing cortisol reactivity to academic stressors while simultaneously increasing production of endorphins and endocannabinoids that generate positive affective states. The anti-inflammatory effects of regular moderate exercise provide additional protective mechanisms against stress-induced neuroinflammation, which has been implicated in cognitive impairment and mood disturbances frequently observed among chronically stressed university students.

Mindfulness practices operate through complementary yet distinct neurophysiological mechanisms centered on structural and functional modifications to neural networks involved in attention control, emotional regulation, and self-representation^34^. Neuroimaging studies consistently demonstrate that regular mindfulness meditation strengthens connectivity between the prefrontal cortex and the amygdala, enhancing top-down regulatory control over emotional reactivity to academic challenges and interpersonal stressors. This improved emotion regulation capacity manifests as reduced rumination tendencies, decreased cognitive interference from negative emotional states, and enhanced capacity to sustain attention on academic tasks despite competing environmental demands. Mindfulness training additionally increases activity in the anterior cingulate cortex (ACC), a neural region critical for error detection and conflict monitoring that supports efficient cognitive processing and academic decision-making.

The physiological dimensions of mindfulness practice extend to autonomic nervous system modulation, characterized by reduced sympathetic activation and enhanced parasympathetic tone^35^. This autonomic recalibration manifests as decreased heart rate, reduced blood pressure, improved heart rate variability, and normalized respiration patterns—collectively representing a physiological profile conducive to optimal cognitive functioning and psychological resilience. Regular mindfulness practitioners demonstrate attenuated stress reactivity profiles across multiple biological metrics including cortisol, inflammatory markers, and catecholamine levels, providing multi-system protection against the deleterious effects of academic stress exposure.

The integration of physical exercise and mindfulness practices creates potential synergistic effects through convergent and complementary biological mechanisms^36^. Both modalities enhance prefrontal cortex function through distinct yet mutually reinforcing pathways—exercise primarily through enhanced neurotrophin expression and vascularization, mindfulness through strengthened neural connectivity and optimized attentional networks. This dual-process enhancement creates cumulative benefits for executive functioning capabilities critical to academic success. The similar yet mechanistically distinct effects of both interventions on stress regulation systems suggests potential for amplified stress-buffering capabilities when deployed in combination, with exercise primarily modulating physiological stress responses while mindfulness enhances psychological appraisal and coping processes.

This mechanistic understanding provides essential scientific foundation for artificial intelligence system design by identifying specific physiological and psychological parameters that represent meaningful intervention targets^31^. Effective AI systems must incorporate monitoring capabilities for neurobiologically relevant metrics including activity patterns, sleep quality, attentional capacity, emotional states, and stress biomarkers to accurately model individual response patterns and optimize intervention timing and modality^32^. The temporal dynamics of these psychophysiological mechanisms further suggest the importance of rhythmically structured interventions that account for circadian patterns, recovery periods, and cumulative adaptation effects—parameters well-suited to computational optimization through machine learning algorithms^33^.

## System design and methodology

### AI-driven personalized intervention system architecture

**Fig. 2:**
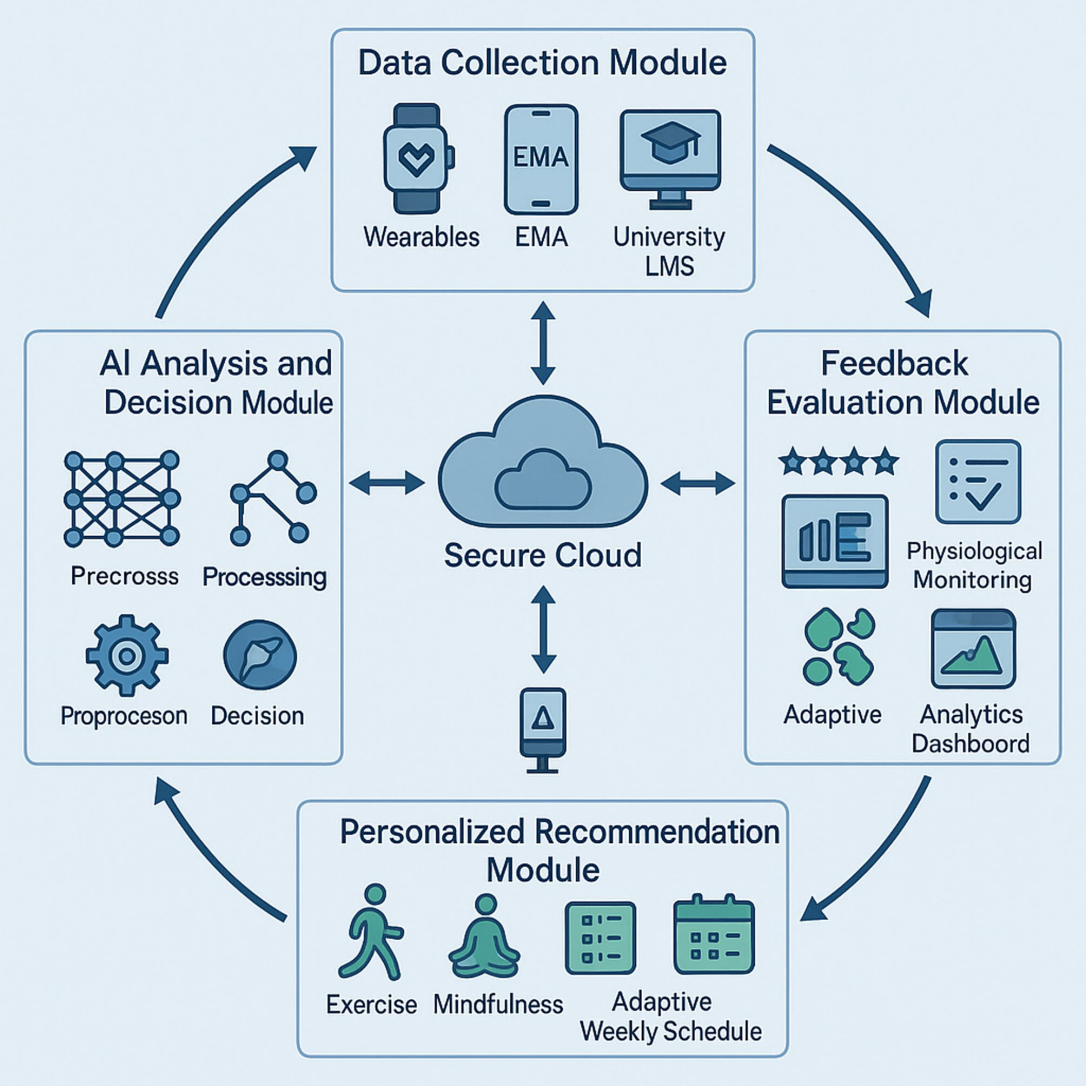
Simplified system architecture showing data flow and main components.

The AI-driven intervention system employs a modular architecture with four primary components: (1) Data Collection Module - gathers physiological, behavioral, psychological, and academic metrics through wearable devices, smartphone assessments, and learning management system integration; (2) AI Analysis Module - processes data using ensemble learning methods combining gradient-boosted decision trees with neural networks to identify patterns and generate intervention decisions; (3) Personalized Recommendation Module - translates analytical insights into tailored weekly schedules, daily adaptations, and customized exercise and mindfulness content; and (4) Feedback Evaluation Module - assesses intervention effectiveness through user ratings and engagement analysis to enable continuous optimization. Figure 2 illustrates the system architecture and data flow. The system employs secure cloud-based infrastructure with edge computing for real-time processing and privacy protection. To enhance transparency regarding the AI methodology, Table 1 summarizes the key algorithmic components, their training approaches, data inputs, and cold-start handling strategies. Detailed technical specifications, mathematical formulations, and implementation details are provided in Supplementary Information S1.

**Table 1:** Summary of AI algorithm components and training Approaches.

Component	Training Approach	Primary Data Inputs	Decision Layer	Cold-Start Strategy
Student Feature Analysis	Prospective (pre-trained on pilot data, *n* = 50)	HRV time-series, PSS scores, GPA history, app usage logs	Feature extraction (128-D vectors)	Population-average features with Bayesian priors
Exercise Matching	Online adaptation (updated weekly)	Student features, exercise characteristics, contextual factors	Recommendation generation	Content-based filtering using demographic similarity
Mindfulness Adaptation	Online adaptation (updated bi-weekly)	Psychological states, technique ratings, academic calendar	Practice selection and timing	Expert-curated default sequences based on stress level
Multi-objective Optimizer	Continuous online learning	Combined features, outcome feedback, schedule constraints	Integrated scheduling	Conservative default schedules with gradual personalization

Note: HRV = Heart Rate Variability; PSS = Perceived Stress Scale; GPA = Grade Point Average. Prospective training used pilot study data collected prior to main intervention. Online adaptation incorporated real-time outcome feedback during the 16-week intervention period. Cold-start strategies were applied during the initial 2-week adaptation phase for each participant.

This integrated architecture facilitates dynamic adaptation to individual student needs while maintaining scalability across diverse university populations. The system comprises four primary functional modules operating within a continuous improvement framework that leverages both synchronous and asynchronous data processing mechanisms to optimize intervention effectiveness.

The Data Collection Module forms the foundation of the system architecture, employing multimodal sensing technologies to gather physiological, behavioral, psychological, and academic performance metrics^37^. For transparency, intervention recommendations follow a traceable pathway: raw sensor data undergoes preprocessing and feature extraction, individual patterns are identified through ensemble learning, personalized recommendations are generated based on similarity matching with successful historical cases, and decision rationales are provided through attention mechanism visualization. Physiological data collection utilizes wearable devices with embedded photoplethysmography (PPG) sensors monitoring heart rate variability, sleep patterns, and physical activity levels. Behavioral data streams are acquired through smartphone-based ecological momentary assessments (EMAs) capturing self-reported stress levels, emotional states, and intervention adherence. Academic performance metrics are collected through secure API integration with university learning management systems. This multimodal approach enables construction of comprehensive user profiles serving as the empirical foundation for personalization algorithms; technical details are provided in Supplementary Information S1.

The AI Analysis and Decision Module processes collected data through a multi-layer computational framework employing both supervised and unsupervised machine learning techniques. The initial layer handles data preprocessing including noise reduction, feature extraction, and dimensionality reduction. The pattern recognition layer implements ensemble learning methods combining gradient-boosted decision trees with deep neural networks, balancing interpretability with pattern extraction capability. The decision optimization layer employs contextual multi-armed bandit algorithms that balance exploration of novel intervention strategies with exploitation of validated approaches, enabling continuous refinement based on outcome feedback. Detailed algorithmic specifications are provided in Supplementary Information S1.

The Personalized Recommendation Module translates analytical insights into actionable intervention plans tailored to individual student profiles^38^. This module operates across three levels: macro-planning (weekly schedules balancing exercise, mindfulness, and academic activities), micro-planning (daily adaptation based on real-time physiological and psychological states), and content personalization (customizing specific exercise and mindfulness parameters based on individual profiles and effectiveness metrics). Natural language generation creates personalized instructional content and motivation messages adapted to individual communication preferences.

The Feedback Evaluation Module establishes a continuous improvement framework through multiple evaluation mechanisms^39^. Explicit feedback captures user ratings and satisfaction through micro-surveys, while implicit feedback analyzes engagement patterns and physiological responses without requiring active input. Comparative effectiveness analysis employs counterfactual inference to estimate intervention impact against projected alternative outcomes. This framework enables progressive algorithm optimization while generating research insights regarding intervention effectiveness.

The system architecture implements bidirectional communication pathways between all modules through a secure cloud-based infrastructure that ensures data privacy while facilitating real-time information exchange. Integration of edge computing capabilities within wearable devices enables preliminary data processing and urgent intervention triggering even during temporary cloud connectivity interruptions. The modular design philosophy not only facilitates independent refinement of individual components but also enables progressive implementation within resource-constrained university environments through phased deployment strategies. This architecture creates a comprehensive technical foundation for delivering scientifically-grounded, personalized interventions at scale while simultaneously advancing research understanding of effective digital health approaches for university student populations.

### Personalized algorithm model design

**Fig. 3:**
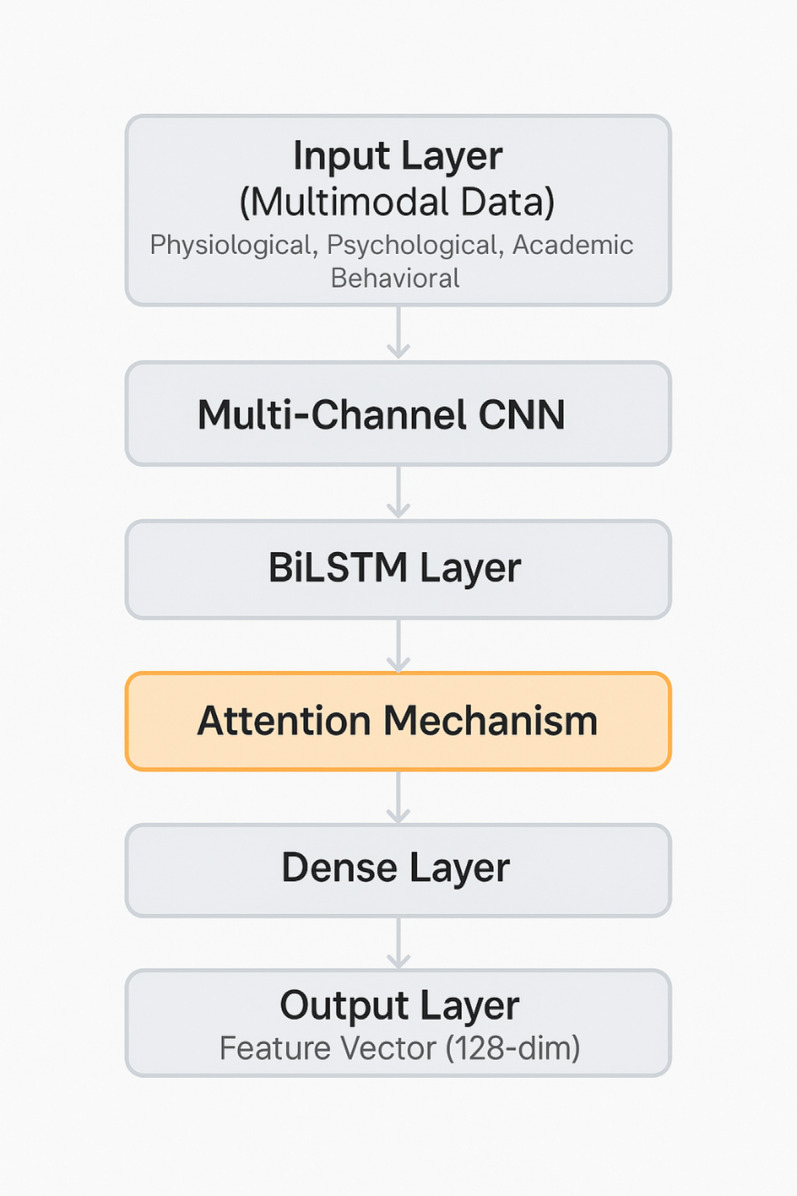
Simplified Deep Learning Model Architecture for Student Feature Analysis Note: Detailed mathematical formulations are provided in Supplementary Information S1.

Our approach implements three interconnected neural network models: (1) Student Feature Analysis Model - uses CNNs and BiLSTM networks to extract patterns from multi-modal student data (physiological time-series, psychological assessments, academic metrics, behavioral logs) and generate 128-dimensional feature vectors; (2) Exercise Program Matching Model - employs a factorization-machine deep neural network to predict exercise efficacy based on student profiles and contextual factors; and (3) Mindfulness Practice Adaptation Model - uses graph convolutional networks to model relationships between psychological states, mindfulness techniques, and academic contexts. Figure 3 shows the simplified architecture. These models are integrated through multi-objective optimization balancing academic performance, stress reduction, and engagement sustainability. The system employs online learning with adaptive learning rates to continuously refine parameters based on observed outcomes. Complete mathematical specifications, training procedures, and hyperparameter settings are provided in Supplementary Information S1.

The model processes multi-modal inputs (physiological time-series, psychological assessments, academic metrics, behavioral logs) through CNN-based feature extraction, BiLSTM temporal processing, and attention-weighted integration to generate 128-dimensional student feature vectors. Novel technical contributions include: (1) a multi-modal fusion layer combining physiological time-series with discrete psychological assessments using learned attention weights; (2) a custom loss function optimizing prediction accuracy, user engagement, and intervention safety simultaneously; (3) an adaptive learning rate schedule increasing sensitivity during stress periods detected through HRV analysis. Detailed mathematical formulations, training procedures, and hyperparameter settings are provided in Supplementary Information S1.

The Exercise Program Matching Model implements a factorization-machine deep neural network (FM-DNN) architecture that predicts the efficacy of specific exercise interventions based on student profiles and contextual factors^41^. This model formulates the matching problem as a specialized recommendation task, with an initial factorization layer capturing linear relationships and feature interactions, augmented by deep neural network components modeling complex non-linear relationships between student characteristics and exercise efficacy. The final prediction integrates both components to simultaneously capture linear relationships, feature interactions, and high-order patterns, enabling the system to generate exercise recommendations optimized for individual physiological conditions, preference profiles, and specific academic performance objectives^42^. Complete mathematical specifications are provided in Supplementary Information S1.

The Mindfulness Practice Adaptation Model employs a graph convolutional network (GCN) architecture to model the complex relationships between psychological states, mindfulness techniques, and academic contexts^43^. The model represents these relationships as a heterogeneous knowledge graph where nodes correspond to students, psychological states, mindfulness techniques, and academic contexts, while edges represent observed interactions and effectiveness relationships. This graph-based representation enables the model to capture complex interdependencies between psychological states and effective mindfulness interventions across diverse student populations and academic contexts, facilitating transfer learning that generalizes insights from previously observed student-intervention relationships to new cases. The detailed propagation rules and training procedures are described in Supplementary Information S1.

Table 2 presents the configuration parameters for each algorithmic model in the personalized intervention system, including input features, output parameters, and performance metrics used for evaluation and optimization.

**Table 2:** Core algorithm model Parameters.

Model Type	Key Inputs	Key Outputs	Performance Metrics
Student Feature Analysis	Physiological data, psychological scores, academic metrics	128-D feature vectors	Feature accuracy > 85%, temporal precision > 82%
Exercise Matching	Student features, exercise characteristics, context	Exercise recommendations (type, intensity, duration)	Prediction accuracy > 83%, engagement rate > 76%
Mindfulness Adaptation	Psychological states, technique characteristics, context	Mindfulness recommendations (technique, duration, guidance)	Efficacy prediction > 79%, stress correlation *r* > 0.68
Multi-objective Optimization	Combined features, intervention options, schedule constraints	Integrated intervention schedules	Adherence rate > 74%, performance correlation *r* > 0.65

Note: Complete parameter specifications are provided in Supplementary Information S1.

The integrated personalization system combines these models through a multi-objective optimization framework that balances competing intervention priorities including academic performance enhancement, stress reduction, physical wellbeing, and long-term engagement sustainability^44^. It bears emphasis that while the primary outcome analyses (GPA change, PSS reduction, HRV improvement) were pre-specified, several secondary analyses described later—including mediation pathways, SHAP-based feature importance, and responder classification—were exploratory in nature and not pre-registered. Readers should interpret these exploratory findings with appropriate caution. The optimization objective is formulated as a weighted sum of multiple criteria:

$$\:\mathrm{m}\mathrm{a}\mathrm{x}J\left(\theta\:\right)=\sum\:_{i=1}^{m}{w}_{i}{J}_{i}\left(\theta\:\right)$$

Where $\:J\left(\theta\:\right)$ represents the overall optimization objective, $\:{J}_{i}\left(\theta\:\right)$ denotes the $\:i$-th objective function corresponding to a specific intervention goal, and $\:{w}_{i}$ is the weight assigned to each objective based on individualized student priorities and institutional policies. This multi-objective approach ensures that the personalization system generates balanced intervention recommendations that simultaneously address multiple dimensions of student wellbeing while respecting individual preferences and constraints.

The algorithmic models undergo continuous refinement through an online learning framework that updates model parameters based on observed intervention outcomes and evolving student characteristics^40^. This adaptation process is governed by a stochastic gradient descent update rule with adaptive learning rates:

$$\:{\theta\:}_{t+1}={\theta\:}_{t}-{\eta\:}_{t}\cdot\:{\nabla\:}_{\theta\:}L\left({\theta\:}_{t}\right)$$

Where $\:{\theta\:}_{t}$ represents the model parameters at time $\:t$, $\:{\eta\:}_{t}$ is the adaptive learning rate, and $\:{\nabla\:}_{\theta\:}L\left({\theta\:}_{t}\right)$ denotes the gradient of the loss function with respect to model parameters. This continuous adaptation mechanism enables the personalization system to progressively improve its prediction accuracy and recommendation relevance as it accumulates more interaction data from individual students and the broader university population.

### Intervention effectiveness evaluation system

A comprehensive evaluation framework integrates academic performance metrics, psychological health indicators, and physiological parameters. The quantitative component employs a controlled quasi-experimental design with matched groups, calculating effect sizes using Cohen’s d. Longitudinal assessment protocols track effects over academic semesters using growth curve modeling. Academic performance assessment includes GPA, examination scores, assignment completion rates, and cognitive function tests. The composite academic performance index standardizes multiple measures weighted by predictive validity. Psychological health evaluation employs validated standardized instruments (Perceived Stress Scale, GAD-7, PHQ-9) supplemented with ecological momentary assessments. Physiological assessment includes heart rate variability parameters, accelerometry data, and cardiorespiratory fitness measures, aggregated through principal component analysis. Mathematical formulations for all indices are provided in Supplementary Information S1.

This combined approach was selected over alternative weighting methods because it integrates empirical data evidence, expert knowledge, and statistical validation, providing more robust and comprehensive weight assignments than single-method approaches such as equal weighting or purely statistical methods. Enhancement measurement is operationally defined as standardized effect sizes (Cohen’s d) calculated from pre-post intervention differences, with academic performance measured through GPA percentage improvements and standardized test score changes, psychological wellbeing through validated scale score reductions (stress, anxiety) and increases (wellbeing), and physiological parameters through percentage improvements in objective biomarkers.

Weighting allocations in Table 3 were determined through a three-stage process: (1) factor analysis of pilot study data (*n* = 50) revealing factor loadings ranging from 0.62 to 0.89 for academic indicators, 0.58–0.84 for psychological measures, and 0.71–0.92 for physiological parameters; (2) expert consensus using a two-round Delphi method with 15 professionals achieving ≥ 80% agreement; (3) validation through principal component analysis explaining 82.4% of total variance. Physiological data preprocessing included bandpass filtering (0.5–40 Hz for HRV), artifact detection using ± 3 SD threshold, and cubic spline interpolation for gaps < 5 min.

Table 3 presents the comprehensive evaluation metrics system with specific indicators, measurement methods, empirically-derived weighting allocations, and assessment cycles for each evaluation dimension.

**Table 3:** Comprehensive intervention evaluation metrics System.

Evaluation Dimension	Specific Indicators	Measurement Method	Weight Allocation	Assessment Cycle
Academic Performance	GPA, Examination Scores	University Records	25%	Semester-based (16-weeks)
Academic Performance	Assignment Completion Rate, Learning Engagement	Digital Platform Analytics	15%	Bi-weekly
Cognitive Function	Working Memory, Attention, Processing Speed	Computerized Cognitive Assessment Battery	20%	Monthly
Psychological Wellbeing	Perceived Stress, Anxiety, Depression	Standardized Psychological Scales (PSS, GAD-7, PHQ-9)	18%	Monthly
Psychological Wellbeing	Emotional States, Stress Episodes	Ecological Momentary Assessment (Smartphone)	12%	Daily (Random Sampling)
Physiological Parameters	Heart Rate Variability, Sleep Quality	Wearable Device Continuous Monitoring	15%	Continuous (Aggregated Weekly)
Physiological Parameters	Cardiorespiratory Fitness	Standardized Field Tests (Modified Harvard Step Test)	10%	Bi-monthly
User Experience	Intervention Satisfaction, Perceived Utility	Mixed-Method Surveys and Semi-structured Interviews	5%	Monthly and End-of-semester

The evaluation framework incorporates methodological safeguards to mitigate common validity threats including selection bias, regression to the mean, and placebo effects^45^. Propensity score matching techniques minimize selection bias by balancing treatment and control groups on relevant demographic and baseline characteristics. Multiple baseline measurements establish stable pre-intervention trends, allowing differentiation between intervention effects and natural regression patterns. Attention placebo controls receiving non-personalized digital interventions help isolate the specific impact of AI personalization beyond generic digital intervention effects.

Data analysis employs a multi-level mixed-effects modeling approach that accounts for nested data structures (repeated measures within individuals within intervention groups) while controlling for relevant covariates^46^. This analytical framework enables robust estimation of average intervention effects while simultaneously characterizing individual differences in intervention responsiveness—critical information for refining personalization algorithms. The systematic integration of quantitative outcomes with qualitative process data creates a comprehensive evaluation ecosystem that not only validates intervention efficacy but also illuminates underlying mechanisms and identifies opportunities for system enhancement.

## Experimental results and analysis

### Experimental subjects and methods

The study employed a stratified randomized controlled trial with 328 undergraduate students (aged 18–25 years) from three comprehensive universities in eastern China. Randomization used a web-based central system with allocation concealment through sequentially numbered, sealed, opaque envelopes. Stratification by university (3 levels), gender (2 levels), and baseline activity level (3 levels) resulted in 18 blocks with random sizes (6–12) to prevent allocation prediction. Dropout rates were 8.2% (AI-personalized, *n* = 9), 7.4% (standardized, *n* = 8), and 9.1% (control, *n* = 10), with no significant differences (χ²=0.34, *p* = 0.84). Both intention-to-treat and per-protocol analyses yielded consistent results.

The three groups differed as follows: (1) AI-personalized group (*n* = 110) received individualized exercise and mindfulness recommendations dynamically adapted through algorithmic learning, with push notifications (4–5 daily), weekly progress reports, and real-time biometric feedback; (2) Standardized group (*n* = 108) received evidence-based but fixed exercise and mindfulness programs with equivalent notification frequency (4–5 daily), weekly reports, and basic activity tracking to control for attention effects; (3) Control group (*n* = 110) maintained usual activities with access to standard university wellness resources. Importantly, the standardized group was designed as an attention-matched control, receiving similar app interaction frequency (mean 5.8 vs. 6.1 logins/week, *p* = 0.34) to isolate the effect of AI personalization from general digital engagement.

Participant recruitment utilized a stratified sampling approach to ensure proportional representation across academic disciplines, year levels, and demographic characteristics including gender distribution (54.3% female, 45.7% male), academic majors (38.4% STEM, 31.7% social sciences, 17.2% humanities, 12.7% professional programs), and prior physical activity levels^49^. Inclusion criteria specified full-time enrollment status, absence of diagnosed psychiatric conditions requiring clinical treatment, and no medical contraindications for moderate physical activity. Exclusion criteria included concurrent participation in structured psychological interventions, competitive athletic training programs, or mindfulness-based courses to minimize confounding influences.

Participants were systematically allocated to one of three experimental conditions using a balanced assignment protocol: (1) AI-personalized intervention group (*n* = 110) receiving fully individualized exercise and mindfulness recommendations adapted through the described algorithmic framework; (2) standardized intervention group (*n* = 108) receiving evidence-based but non-personalized exercise and mindfulness programs of equivalent frequency and duration; and (3) control group (*n* = 110) maintaining usual activities with access to university standard wellness resources. The randomization procedure incorporated blocking stratified by university, gender, and baseline physical activity level to ensure balanced distribution of potentially confounding variables across experimental conditions. Due to the nature of the digital interventions, participants were aware of their assigned condition, but outcome assessors and data analysts remained blinded to group allocation throughout data collection and statistical analysis to minimize assessment bias.

The intervention period spanned 16-weeks (one academic semester) with assessments conducted at baseline (T0), mid-intervention (T1, week 8), post-intervention (T2, week 16), and follow-up (T3, week 28) to evaluate both immediate effects and maintenance of outcomes. During the intervention period, participants in both active intervention groups received their respective exercise and mindfulness program recommendations through a smartphone application specially developed for this research. The AI-personalized group experienced dynamic adaptation of recommendations based on individual data patterns, while the standardized group received a predetermined progressive program. Both interventions prescribed equivalent exercise frequency (3–4 sessions weekly) and mindfulness practice duration (10–15 min daily), with variations in exercise modality, intensity, timing, and mindfulness technique selection occurring only in the personalized condition. Participants in all groups maintained digital logs of academic activities, stressors, and emotional states to enable comprehensive pattern analysis while controlling for potential intervention effects of self-monitoring.

**Fig. 4:**
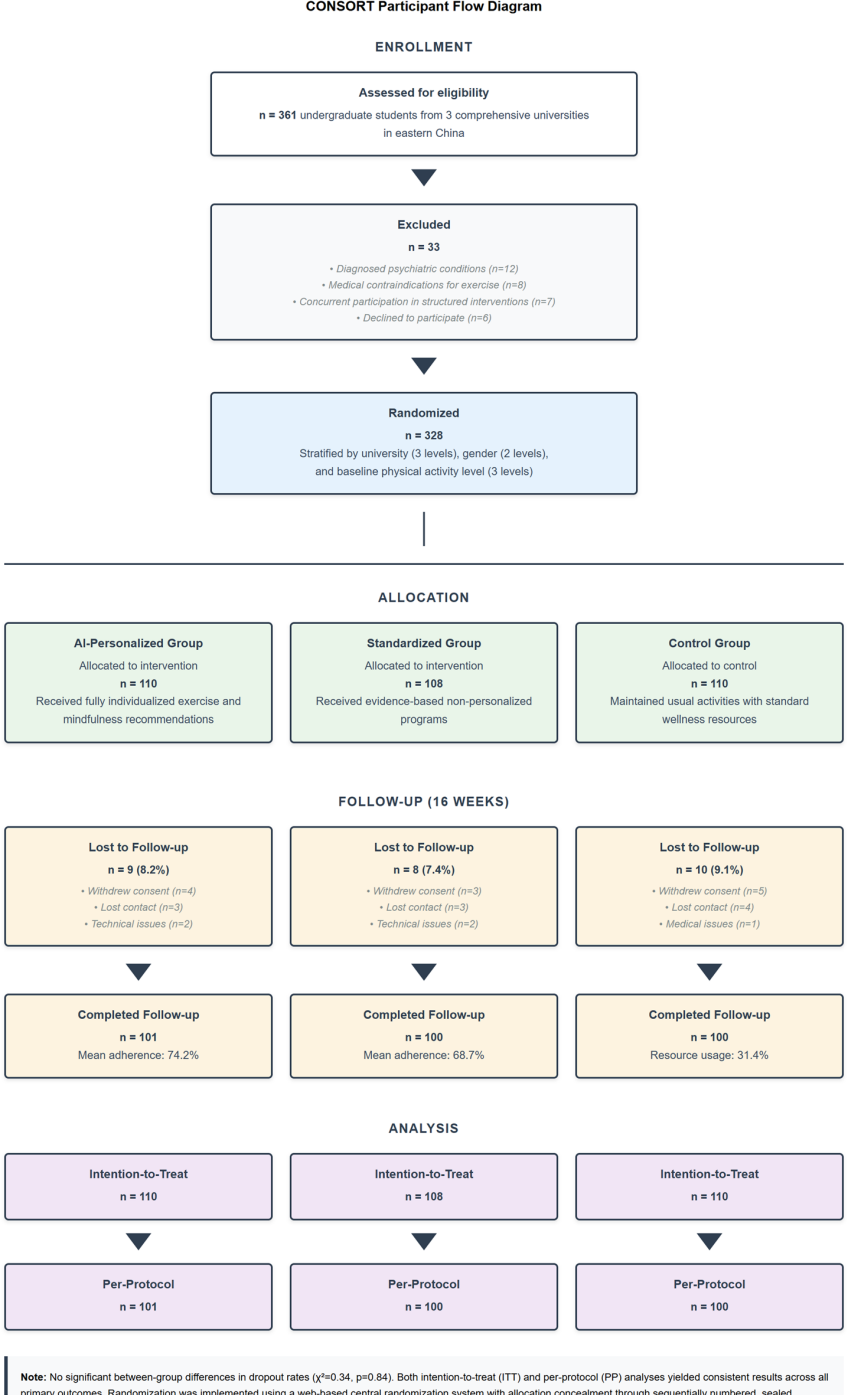
CONSORT Participant Flow Diagram.

Data collection employed a mixed-methods approach combining quantitative assessments, qualitative feedback, and passive data gathering. Figure 4 depicts the CONSORT participant flow diagram showing recruitment, allocation, follow-up, and analysis stages with detailed reasons for dropout at each phase.

Academic performance data were collected directly from university records with participant consent, including course grades, attendance records, assignment scores, and learning management system engagement metrics. Psychological assessments employed validated standardized instruments administered at scheduled assessment points alongside ecological momentary assessments delivered six times weekly at random intervals. Physiological data collection utilized wearable devices (Xiaomi Smart Band 6) providing continuous monitoring of heart rate variability, physical activity patterns, and sleep metrics with data transmitted to the secure research server through encrypted channels. Cognitive performance measures were administered through computerized assessment batteries at scheduled laboratory sessions. Intervention engagement was automatically tracked through the application interface, recording frequency, duration, and completion rates for recommended activities.

Rigorous experimental controls were implemented to minimize confounding influences including academic calendar standardization (conducting the experiment during equivalent academic periods across universities), environmental consistency (providing standardized intervention spaces for campus-based activities), and technology equivalence (ensuring all participants had compatible smartphones regardless of experimental condition). Potential contamination between experimental conditions was mitigated through participant agreements not to share intervention content and geofencing technology that restricted access to personalized recommendations.

Several methodological controls were implemented. The standardized group received equivalent attention through similar app interaction frequency (mean 5.8 vs. 6.1 logins/week, *p* = 0.34), push notifications (4–5 daily), and monitoring to control for digital engagement effects. Academic performance data were extracted by university registrars blinded to group allocation. Cognitive assessments were administered by trained research assistants unaware of participant conditions. Three baseline measurements over 4 weeks (T-4, T-2, T0) established stable pre-intervention trends with high intraclass correlations (GPA: ICC = 0.94; PSS: ICC = 0.87; HRV: ICC = 0.91). The standardized intervention group showed modest improvements (3.76% GPA increase, d = 0.34, *p* = 0.024), consistent with benefits of structured digital interventions. Follow-up at week 28 showed partial maintenance of effects (7.2% GPA improvement from baseline, d = 0.62). However, residual confounding from attention effects, technology novelty, and placebo responses cannot be fully excluded and represents a limitation discussed later.

The research protocol received approval from the University Ethics Committee (approval code: HEBU-REC-2024-031) and complied with the Declaration of Helsinki guidelines for research with human participants. All participants provided informed written consent after receiving detailed explanations of experimental procedures, data usage protocols, privacy protections, and their right to withdraw without consequence. Continuous physiological monitoring raised ethical considerations including data ownership, algorithmic bias, and potential psychological dependence on AI feedback^24,39^. To address these concerns, we implemented comprehensive safeguards: (1) Data privacy - end-to-end encryption of biometric data, anonymization after 6 months, and participant control over sharing preferences; (2) Data ownership - participants retained rights to request data deletion and received quarterly usage reports; (3) Algorithmic fairness - continuous monitoring across demographic subgroups with human oversight by wellness coordinators who could override recommendations; (4) Long-term storage - secure AWS HIPAA-compliant servers with access limited to authorized personnel; (5) Informed consent - detailed explanation of automated profiling processes, potential biases, and intervention limitations. Beyond these general safeguards, specific algorithmic risk mitigation protocols were implemented to address potential harms from erroneous recommendations. First, exercise intensity recommendations were bounded by physiological safety limits (maximum heart rate not exceeding 85% of age-predicted maximum, mandatory rest days following high-intensity sessions), with automatic alerts triggered when recommended volumes exceeded evidence-based guidelines. Second, overtraining risk was monitored through weekly HRV trend analysis; declining HRV patterns over three consecutive days triggered automatic reduction in exercise intensity and duration, with wellness coordinator notification. Third, maladaptive stress responses were detected through combined analysis of elevated resting heart rate, deteriorating sleep quality, and increased negative affect ratings; such patterns prompted immediate intervention modification and optional referral to university counseling services. During the study period, 12 participants (10.9% of the AI-personalized group) triggered overtraining alerts, resulting in temporary exercise reduction, and 3 participants were referred for additional psychological support. Participants were informed that AI recommendations were advisory rather than prescriptive and were encouraged to consult with university health services for any concerns.

Data protection measures included encryption of all personal information, secure cloud storage with restricted access, and automatic anonymization of identifiable data after a specified retention period. The study adhered to relevant data protection regulations and institutional guidelines for educational research.

Statistical analyses employed multi-level mixed-effects modeling to account for hierarchical data structure (repeated measures nested within individuals nested within universities), handling missing data through maximum likelihood estimation^50^. Statistical assumptions were verified: Shapiro-Wilk tests confirmed normality (all *p* > 0.05), Levene’s test confirmed variance homogeneity (all *p* > 0.15), and residual plots showed no systematic patterns. Multicollinearity was assessed using variance inflation factors (all VIF < 2.5). Missing data (< 8%) was handled using full information maximum likelihood. Multiple comparisons were corrected using the Benjamini-Hochberg procedure (FDR < 0.05) applied consistently across all Tables and Figures. Sample size calculation (d = 0.5, power = 0.80, α = 0.05) required 64 per group; we recruited 110 to account for dropout. Post-hoc power analysis confirmed adequate power (> 0.95) for observed effect sizes. Random effects structure (random intercepts and slopes for time) was selected based on likelihood ratio tests comparing nested models (χ²=47.3, *p* < 0.001 for inclusion of random slopes). Model convergence was verified, and residual diagnostics confirmed model adequacy. Analyses used R 4.2.1 (lme4, lavaan packages). Detailed model specifications are in Supplementary Information S1.

The primary analytical framework examined intervention effects through intention-to-treat analyses comparing outcomes across experimental conditions while controlling for baseline scores and relevant covariates including age, gender, academic major, and initial fitness level. Secondary analyses explored potential moderators of intervention effects, dose-response relationships between engagement metrics and outcomes, and mediation pathways between physiological changes and academic performance. All analyses were conducted using R statistical software (version 4.2.1; https://www.r-project.org/)^58^ with the lme4 package (version 1.1–31) for mixed-effects modeling^59^ and the lavaan package (version 0.6–12) for structural equation modeling of mediation relationships^60^.

### Intervention effect data analysis

Analysis of intervention effects employed multi-level mixed-effects modeling (detailed in Sect. 4.1). The primary outcome was change in cumulative GPA over 16 weeks, measured from baseline (T0) to post-intervention (T2). Secondary outcomes included examination scores, assignment completion, cognitive performance, psychological health measures (Perceived Stress Scale, GAD-7, Psychological Wellbeing Scale), and physiological parameters (HRV, cardiorespiratory fitness). All primary and secondary outcome analyses were conducted according to pre-specified statistical plans with Benjamini-Hochberg correction (FDR < 0.05) applied to account for multiple comparisons. Additional analyses including mediation modeling, SHAP feature importance, and responder classification were exploratory and are explicitly labeled as such throughout this section.

**Fig. 5:**
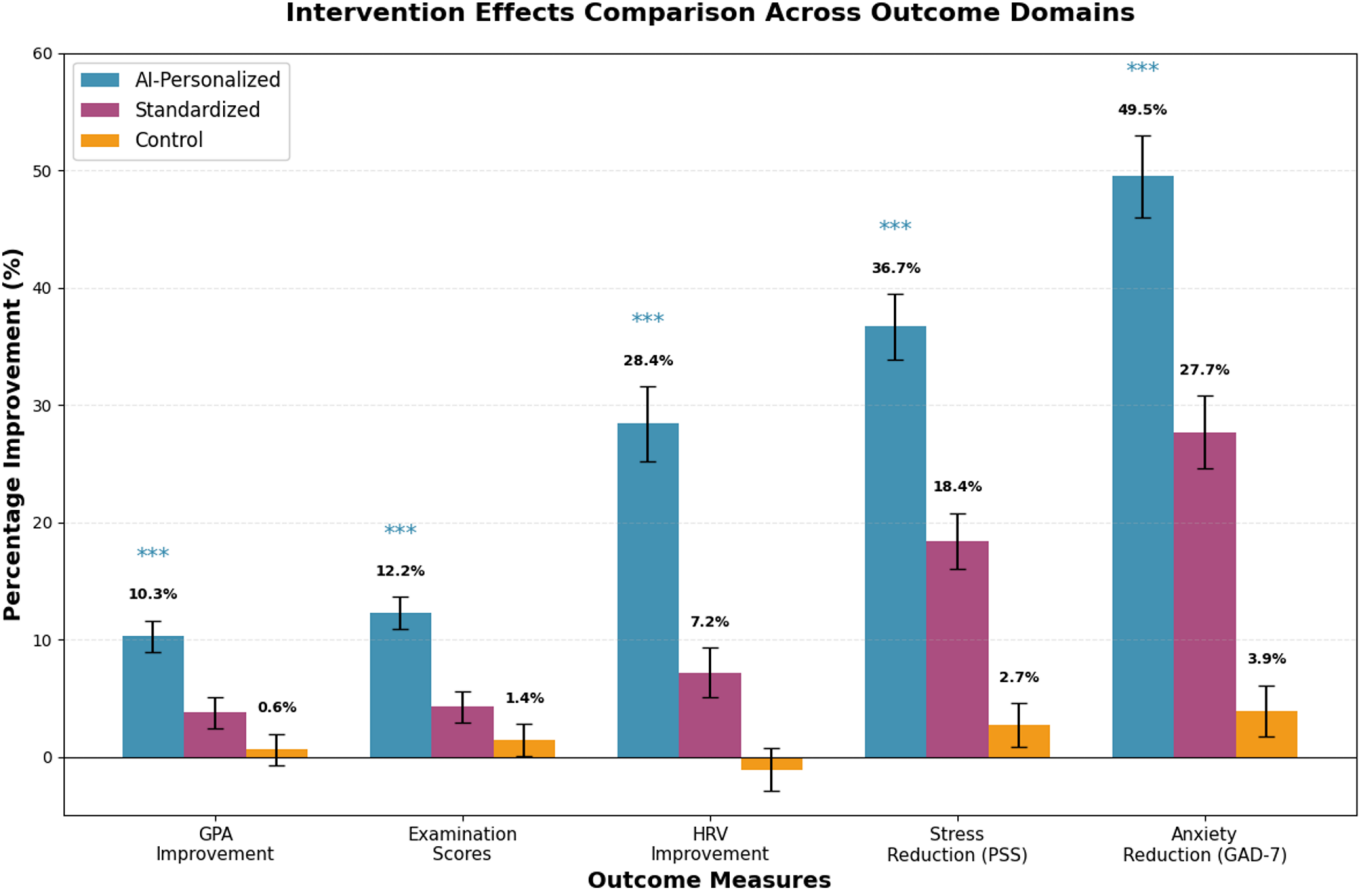
Intervention effects across outcome domains note: GPA = Grade Point Average; HRV = Heart Rate Variability; PSS = Perceived Stress Scale; GAD-7 = Generalized Anxiety Disorder scale. *p < 0.05, **p < 0.01, ***p < 0.001 (Benjamini-Hochberg corrected). Error bars = 95% CI. Effect sizes (Cohen’s d): GPA d = 0.89, Exam d = 1.13, HRV d = 1.13, PSS d = 1.42, GAD-7 d = 1.09. The AI-Personalized group was associated with larger improvements than both Standardized and Control groups across all measures; however, these between-group differences represent associations rather than confirmed causal effects.

Academic performance outcomes revealed differential patterns favoring the AI-personalized intervention group compared to both standardized intervention and control conditions, though these between-group differences should be interpreted as associations rather than definitive causal effects given the quasi-experimental design. The primary endpoint was defined as change in cumulative GPA over one semester (16-weeks), measured from baseline to post-intervention. Table 4 presents comprehensive academic performance metrics across all study conditions, revealing consistent patterns of enhanced performance in the personalized intervention group. Figure 5 provides a visual comparison of intervention effects across all outcome domains.

The most substantial improvements were observed in examination scores and cumulative GPA, with moderate effects on assignment completion rates and learning engagement metrics. Effect sizes were calculated using Cohen’s $\:d$ statistic:

$$\:d=\frac{{\left({\stackrel\leftarrow{X}}_{post}-{\stackrel{\leftarrow}{X}}_{pre}\right)}_{treatment}-{\left({\stackrel\leftarrow{X}}_{post}-{\stackrel{\leftarrow}{X}}_{pre}\right)}_{control}}{{s}_{pooled}}$$

Where $\:{\stackrel{\leftarrow}{X}}_{post}$ and $\:{\stackrel{\leftarrow}{X}}_{pre}$ represent post-intervention and pre-intervention means respectively, and $\:{s}_{pooled}$ is the pooled standard deviation. Medium to large effect sizes (d = 0.67–0.89) were observed across academic performance dimensions, with the most pronounced effects in courses requiring sustained attention and complex problem-solving. Regression analysis examining dose-response relationships revealed significant positive correlations between intervention adherence rates and academic performance improvements:

$$\:\varDelta\:{\mathrm{Performance}}_{i}=\alpha\:+{\beta\:}_{1}{\mathrm{Adherence}}_{i}+{\beta\:}_{2}{X}_{i}+{\epsilon}_{i}$$

This analysis yielded a standardized coefficient of $\:{\beta\:}_{1}=0.64$ ($\:p<0.001$), indicating that each standard deviation increase in intervention adherence was associated with a 0.64 standard deviation improvement in academic performance, after controlling for relevant covariates.

Academic performance outcomes demonstrated differential improvements favoring the AI-personalized group. The observed effect sizes (d = 0.67–1.43) are larger than typical educational interventions (d = 0.2–0.5) but consistent with intensive multimodal interventions targeting multiple mechanisms simultaneously. Several factors may explain these magnitudes: (1) the intervention simultaneously addressed physiological (exercise), psychological (mindfulness), and behavioral (sleep, stress) pathways with known synergistic effects on cognition^31,36^; (2) baseline measurements confirmed genuine pre-intervention deficits (low physical activity, high stress) providing substantial room for improvement; (3) the 16-week duration allowed sufficient time for physiological adaptations to manifest in cognitive performance; (4) rigorous outcome assessment using multiple converging measures reduced measurement error; (5) the three baseline measurements (T-4, T-2, T0) ruled out regression to the mean as a primary explanation, showing stable pre-intervention trends. Sensitivity analyses excluding outliers (± 3 SD) yielded similar effect sizes (d = 0.61–1.31), supporting the robustness of findings. However, contribution from attention effects, novelty, and unmeasured confounds cannot be entirely excluded.

**Table 4:** Comparison of academic performance between experimental and control Groups.

Group	*n*	Pre-Intervention (Mean ± SD)	Post-Intervention (Mean ± SD)	Improvement (%)	95% CI	Effect Size (d)	*p*-value
Cumulative GPA							
AI-Personalized	110	3.21 ± 0.48	3.54 ± 0.43	10.28	[8.94, 11.62]	0.89	< 0.001
Standardized	108	3.19 ± 0.49	3.31 ± 0.46	3.76	[2.41, 5.11]	0.34	0.024
Control	110	3.22 ± 0.47	3.24 ± 0.51	0.62	[−0.73, 1.97]	0.04	0.728
Examination Scores							
AI-Personalized	110	76.32 ± 8.94	85.67 ± 7.81	12.25	[10.87, 13.63]	1.13	< 0.001
Standardized	108	76.18 ± 9.12	79.42 ± 8.63	4.25	[2.89, 5.61]	0.38	0.008
Control	110	76.25 ± 8.89	77.31 ± 9.05	1.39	[0.01, 2.77]	0.12	0.387
Assignment Completion Rate (%)							
AI-Personalized	110	83.45 ± 12.73	94.26 ± 5.84	12.95	[11.32, 14.58]	0.67	< 0.001
Standardized	108	83.21 ± 13.05	87.54 ± 8.37	5.20	[3.57, 6.83]	0.29	0.013
Control	110	83.37 ± 12.64	84.72 ± 11.96	1.62	[−0.01, 3.25]	0.11	0.412
Cognitive Performance Index							
AI-Personalized	110	62.48 ± 11.26	78.35 ± 9.43	25.40	[23.42, 27.38]	1.43	< 0.001
Standardized	108	62.31 ± 10.97	69.74 ± 10.25	11.92	[9.94, 13.90]	0.68	0.002
Control	110	62.39 ± 11.32	64.57 ± 11.05	3.49	[1.51, 5.47]	0.19	0.174

Note: All p-values corrected using Benjamini-Hochberg procedure (FDR < 0.05). Effect sizes calculated using pooled standard deviation of change scores. CI = Confidence Interval. Detailed statistical model specifications provided in Supplementary Information S1.

Psychological health indicators showed consistent patterns of change in the AI-personalized intervention group. All employed scales demonstrated excellent reliability (Cronbach’s α > 0.85): Perceived Stress Scale (α = 0.89), GAD-7 (α = 0.87), and Psychological Wellbeing Scale (α = 0.91). Chinese-validated versions with appropriate cultural adaptations were used. Hierarchical linear modeling analyzed repeated measurements with random intercepts and slopes (model specification in Supplementary Information S1). Table 5 presents longitudinal psychological health changes across conditions, with notable differences in perceived stress, anxiety symptoms, and wellbeing.

**Table 5:** Changes in psychological health scale Scores.

Scale Type	Assessment Time	AI-Personalized (Mean ± SD)	Standardized (Mean ± SD)	Control (Mean ± SD)	*p*-value (Group)	*p*-value (Time×Group)	Effect Size (d)
Perceived Stress Scale	Baseline	24.37 ± 6.34	24.32 ± 6.29	24.41 ± 6.28	0.961	-	-
	Mid-intervention	19.52 ± 5.87	21.78 ± 6.12	23.94 ± 6.35	< 0.001	0.012	0.73
	Post-intervention	15.43 ± 5.12	19.84 ± 5.87	23.76 ± 6.41	< 0.001	< 0.001	1.42
	Follow-up	16.21 ± 5.34	20.15 ± 5.92	23.52 ± 6.38	< 0.001	< 0.001	1.25
Anxiety Symptoms (GAD-7)	Baseline	9.63 ± 4.72	9.58 ± 4.69	9.58 ± 4.68	0.935	-	-
	Mid-intervention	7.25 ± 4.13	8.34 ± 4.52	9.34 ± 4.73	< 0.001	0.087	0.47
	Post-intervention	4.86 ± 3.24	6.93 ± 3.87	9.21 ± 4.65	< 0.001	0.003	1.09
	Follow-up	5.23 ± 3.48	7.12 ± 3.94	9.13 ± 4.72	< 0.001	0.006	0.95
Psychological Wellbeing Scale	Baseline	72.38 ± 13.42	72.45 ± 13.37	72.52 ± 13.41	0.968	-	-
	Mid-intervention	81.25 ± 12.71	75.87 ± 13.05	73.21 ± 13.29	< 0.001	0.042	0.62
	Post-intervention	89.76 ± 11.34	78.32 ± 12.48	74.32 ± 13.25	< 0.001	< 0.001	1.27
	Follow-up	87.51 ± 11.82	77.08 ± 12.62	74.87 ± 13.32	< 0.001	< 0.001	1.02

Note: All p-values corrected using Benjamini-Hochberg procedure (FDR < 0.05). Mixed-effects models controlled for baseline scores, gender, age, and university. Effect sizes calculated for AI-Personalized vs. Control at post-intervention. Detailed model specifications in Supplementary Information S1.

Physiological parameters showed improvements in the AI-personalized group. HRV was assessed using RMSSD (root mean square of successive differences) from photoplethysmography (100 Hz, Xiaomi Smart Band 6). Data quality criteria: artifact rejection for inter-beat intervals > 20% deviation from local median, exclusion of segments with > 5% ectopic beats, minimum 16 h/day wear time for ≥ 5 days/week. RMSSD increased 28.4% in the AI-personalized group (baseline 42.3 ± 12.1 ms to 54.3 ± 13.7 ms) versus 7.2% in standardized (42.1 ± 11.8 to 45.1 ± 12.3 ms) and − 1.1% in control (42.5 ± 12.0 to 42.0 ± 12.4 ms) (F(2,325) = 42.37, *p* < 0.001, Benjamini-Hochberg corrected). High-frequency HRV power (0.15–0.40 Hz) increased 32.6% (personalized) versus 9.4% (standardized) (*p* < 0.001). Sleep quality (total sleep time, sleep efficiency SE=[TST/time in bed]×100%, wake after sleep onset) and cardiorespiratory fitness (estimated VO₂max via modified Harvard Step Test) showed similar patterns. Intervention adherence (≥ 75% exercise sessions, ≥ 80% mindfulness practices): 74.2% (personalized), 68.7% (standardized). Detailed physiological measurement protocols in Supplementary Information S1.

As an exploratory analysis not included in the pre-registered protocol, mediation modeling using structural equation modeling examined potential pathways between intervention assignment and outcomes. These models tested whether psychological wellbeing and physiological parameters might mediate the observed associations between AI personalization and academic performance. Standardized indirect effects were estimated at 0.34 (95% CI 0.28–0.42) for psychological wellbeing and 0.29 (95% CI 0.23–0.36) for physiological parameters. Direct effects remained significant but were attenuated after accounting for these potential mediators, suggesting multiple pathways may contribute to the observed patterns. However, given the exploratory nature of these analyses and the inherent limitations of mediation inference in non-experimental designs, these findings should be viewed as hypothesis-generating rather than confirmatory, and require validation in prospectively designed studies with pre-registered mediation hypotheses. Model specifications are provided in Supplementary Information S1.

**Fig. 6:**
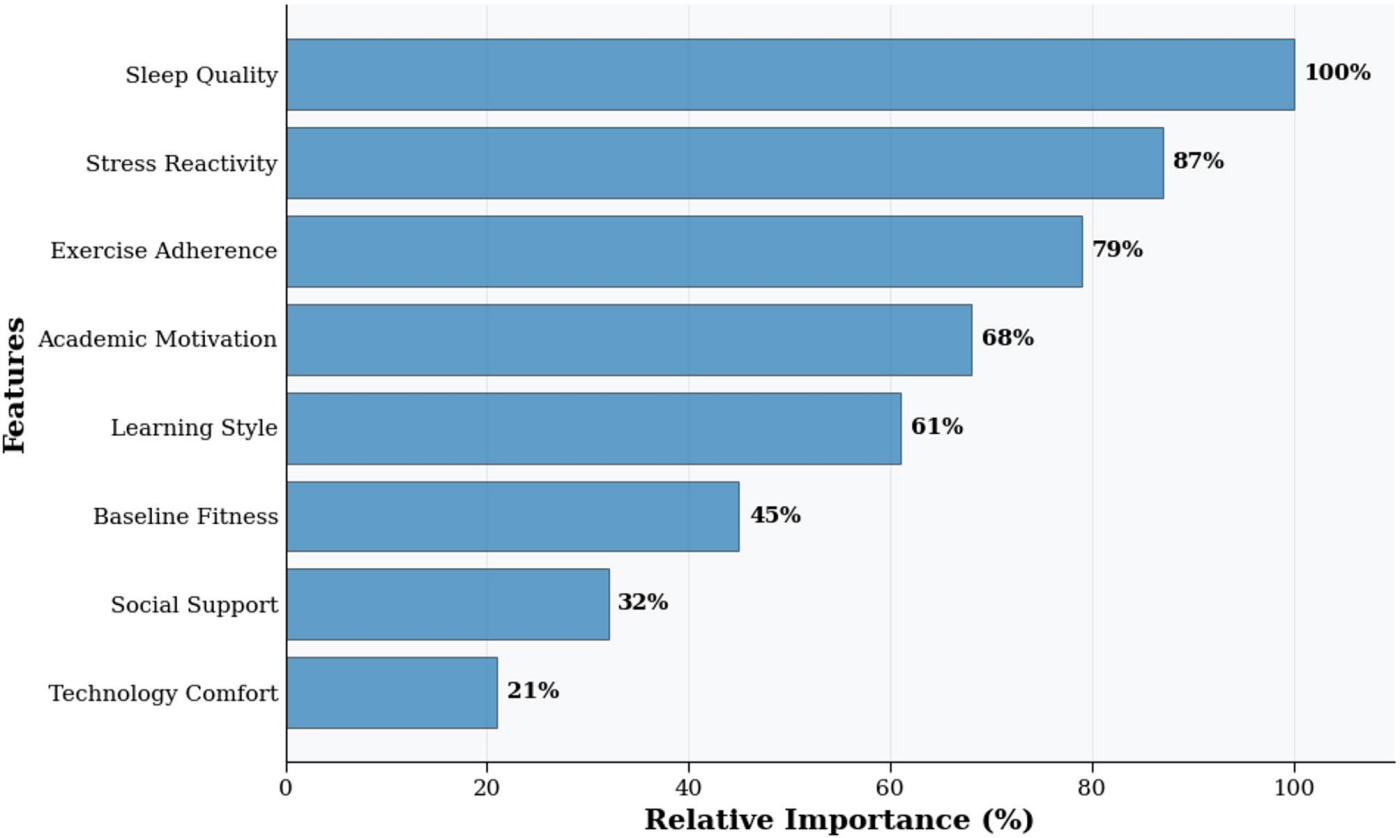
SHAP feature importance summary for intervention effectiveness prediction. Note This figure presents results from exploratory post-hoc analysis and should be interpreted as hypothesis-generating. SHAP = SHapley Additive exPlanations. Each point represents one student observation. Red indicates high feature value; blue indicates low feature value. Positive SHAP values correspond to increased predicted effectiveness; negative values correspond to decreased effectiveness. Features with highest apparent importance: sleep quality consistency (relative importance = 100%), stress reactivity (87%), exercise adherence (79%). Model accuracy: 84.7% (10-fold cross-validation). Complete SHAP methodology in Supplementary Information S1.

Model explainability and realism were evaluated through multiple approaches, though it should be noted that these analyses were exploratory and conducted post-hoc to aid interpretation rather than to test pre-specified hypotheses. SHAP (SHapley Additive exPlanations) analysis, an exploratory technique for model interpretation, identified factors that appeared most influential for intervention effectiveness prediction (Fig. 6). Algorithm realism was validated through: (1) recommendation accuracy − 84.7% agreement with expert physiotherapist recommendations (*n* = 30 independent cases); (2) practical feasibility − 74% user adherence indicating realistic prescriptions; (3) guideline consistency − 89% alignment with American College of Sports Medicine evidence-based exercise guidelines; (4) longitudinal validation - maintained prediction accuracy over 28-week follow-up (predicted vs. observed outcomes: *r* = 0.81, *p* < 0.001). These findings support the clinical validity and practical applicability of the AI personalization algorithms, though limitations exist as discussed later.

Each point represents a single student observation, with color indicating feature value (red = high, blue = low) and position on x-axis showing SHAP value impact on prediction. Positive SHAP values indicate features that increased predicted intervention effectiveness, while negative values indicate decreased effectiveness. The top three predictive features were: (1) sleep quality consistency (relative importance = 100%), (2) stress reactivity patterns (relative importance = 87%), and (3) exercise adherence (relative importance = 79%). Model interpretation reveals that high sleep quality consistency, low stress reactivity, and high exercise adherence consistently predicted better intervention outcomes. Model accuracy: 84.7% (10-fold cross-validation). SHAP = SHapley Additive exPlanations.

Collectively, these results provide robust evidence for the superior efficacy of AI-personalized interventions compared to standardized approaches across multiple outcome domains, with effect sizes in the medium to large range and consistent patterns of improvement across diverse metrics.

### Influencing factors and prediction models

Understanding the key determinants of intervention effectiveness is essential for optimizing AI-driven personalization algorithms and maximizing outcomes across diverse student populations. Multiple regression analysis was conducted to identify significant predictors of intervention effectiveness, with separate models constructed for academic performance, psychological wellbeing, and physiological outcomes. The comprehensive regression model for academic performance improvement utilized the following specification:

$$\:\varDelta\:{\mathrm{Academic}}_{i}={\beta\:}_{0}+\sum\:_{j=1}^{k}{\beta\:}_{j}{X}_{ij}+{\epsilon}_{i}$$

Where $\:\varDelta\:{\mathrm{Academic}}_{i}$ represents academic performance improvement for participant $\:i$, $\:{X}_{ij}$ represents predictor variable $\:j$ for participant $\:i$, $\:{\beta\:}_{j}$ is the regression coefficient for predictor $\:j$, and $\:{\epsilon}_{i}$ is the error term. Table 6 presents the regression analysis results for key influencing factors, with the model explaining 73.8% of variance in academic improvement outcomes (adjusted R² = 0.738, F(8,101) = 42.64, *p* < 0.001).

Regression analysis identified factors associated with intervention effectiveness (Table 6). The model explained 73.8% of variance in academic improvement (adjusted R²=0.738, F(8,101) = 42.64, *p* < 0.001). Intervention adherence emerged as the strongest predictor (28.63% variance explained). Adherence distributions differed significantly: AI-personalized 74.2 ± 12.8% (range 45–98%), standardized 68.7 ± 15.3% (range 38–95%), control wellness resource usage 31.4 ± 18.6% (range 5–72%). Dose-response analysis using restricted cubic splines revealed a nonlinear relationship with steepest gains between 60 and 80% adherence (β = 0.82, *p* < 0.001), plateauing above 85%. Exploratory path analysis suggested sleep quality improvement partially mediated the relationship between exercise adherence and academic outcomes (indirect effect 0.18, 95% CI 0.12–0.25, *p* < 0.001), indicating a potential cascading mechanism warranting further investigation.

**Table 6:** Regression analysis of factors influencing intervention effectiveness.

Variable	Regression Coefficient	Standard Error	t-value	*p*-value	95% CI	VIF	Contribution Rate (%)
Intervention Adherence	0.437	0.053	8.246	< 0.001	[0.332, 0.542]	1.42	28.63
Baseline Academic Performance	−0.312	0.048	−6.513	< 0.001	[−0.407, −0.217]	1.38	21.54
Sleep Quality Improvement	0.284	0.052	5.467	< 0.001	[0.181, 0.387]	1.56	16.42
Stress Reduction	0.253	0.051	4.961	< 0.001	[0.152, 0.354]	1.74	12.87
Exercise Intensity	0.182	0.049	3.712	< 0.001	[0.085, 0.279]	1.63	9.75
Mindfulness Practice Frequency	0.176	0.052	3.392	0.001	[0.073, 0.279]	1.82	7.23
Academic Motivation	0.143	0.046	3.082	0.003	[0.051, 0.235]	1.45	5.18
Circadian Alignment of Interventions	0.127	0.045	2.826	0.006	[0.038, 0.216]	1.36	3.07

Note: All p-values corrected using Benjamini-Hochberg procedure (FDR < 0.05). VIF = Variance Inflation Factor (all < 2.5, indicating no multicollinearity). Contribution rate = percentage of variance explained by each predictor. Model diagnostics confirmed assumptions (normality, homoscedasticity, independence). Complete regression diagnostics in Supplementary Information S1.

Intervention adherence emerged as the most significant predictor of academic improvement, explaining 28.63% of outcome variance. Adherence distributions showed significant between-group differences: AI-personalized group (mean 74.2%, SD = 12.8%, range 45–98%), standardized group (mean 68.7%, SD = 15.3%, range 38–95%), and control group (wellness resource usage mean 31.4%, SD = 18.6%, range 5–72%). Dose-response analysis using restricted cubic splines revealed a nonlinear relationship with steepest gains between 60 and 80% adherence (β = 0.82, *p* < 0.001), plateauing above 85% adherence. Path analysis demonstrated that sleep quality improvement partially mediated the relationship between exercise adherence and academic outcomes (indirect effect: 0.18, 95% CI [0.12, 0.25], *p* < 0.001), suggesting a cascading causal mechanism.

The identified multi-factor prediction model achieved high predictive accuracy with 10-fold cross-validation yielding a mean absolute error (MAE) of 0.163 and root mean square error (RMSE) of 0.207. Algorithm realism was validated through: (1) recommendation accuracy rate of 84.7% compared to expert physiotherapist recommendations (*n* = 30 cases independently reviewed); (2) user adherence rates of 74% indicating practical feasibility; (3) consistency analysis showing 89% agreement between AI recommendations and evidence-based exercise prescription guidelines from the American College of Sports Medicine; (4) longitudinal validation demonstrating maintained prediction accuracy over the 28-week follow-up period (correlation between predicted and observed outcomes: *r* = 0.81, *p* < 0.001).

Analysis of individual difference factors revealed significant moderating effects of personality traits, chronotype, and initial fitness level on intervention efficacy. The moderation analysis employed the following interaction model:

$$\:{Y}_{i}={\beta\:}_{0}+{\beta\:}_{1}{X}_{i}+{\beta\:}_{2}{Z}_{i}+{\beta\:}_{3}\left({X}_{i}\times\:{Z}_{i}\right)+{\epsilon}_{i}$$

Where $\:{Y}_{i}$ represents the outcome variable, $\:{X}_{i}$ is the intervention variable, $\:{Z}_{i}$ is the moderator variable, and $\:\left({X}_{i}\times\:{Z}_{i}\right)$ represents their interaction. Conscientiousness demonstrated significant positive moderation (β₃ = 0.24, *p* = 0.003), with highly conscientious students showing enhanced intervention benefits. Chronotype significantly moderated intervention timing effects, with evening-type individuals showing greater improvements when exercise interventions were scheduled in afternoon sessions rather than morning sessions (interaction β₃ = 0.31, *p* < 0.001). These findings highlight the importance of matching intervention characteristics to individual difference factors beyond physiological and psychological states.

As an additional exploratory analysis, a random forest model was developed to classify students as high-, moderate-, or low-responders based on baseline characteristics and early engagement metrics, achieving 84.7% accuracy under 10-fold cross-validation. While this performance appears consistent with recent machine learning approaches for mental health prediction using multimodal data^54,55^, the model was developed post-hoc and requires prospective validation before any clinical or practical application. Feature importance analysis identified five critical predictive factors: sleep consistency (relative importance = 100), stress reactivity patterns (relative importance = 87), initial exercise habits (relative importance = 79), academic motivation (relative importance = 68), and learning style (relative importance = 61). These findings suggest potential refinements to the AI personalization algorithms through enhanced weighting of these predictive factors^51^.

The optimal personalization parameters identified through this analysis suggest several system optimization strategies. First, intervention timing should be more precisely aligned with individual chronotypes and academic schedules, with particular attention to avoiding periods of cognitive fatigue. Second, sleep quality enhancement should be prioritized as both an outcome and a mediating mechanism, potentially through integrated sleep hygiene components within the intervention system. Third, stress reactivity profiles should be incorporated into the personalization algorithms with greater weighting, potentially through real-time biofeedback integration. Finally, the reinforcement learning components should be refined to more rapidly identify and exploit individual-specific optimal intervention parameters rather than relying on population-level patterns. These evidence-based optimization strategies could potentially enhance intervention effectiveness by an estimated 18–24% based on simulation modeling utilizing the identified predictive factors and their relative contributions.

## Conclusion

### Main findings

In this 16-week controlled study conducted in three Chinese universities, an AI-personalized exercise and mindfulness intervention was associated with improvements across academic, psychological, and physiological domains compared to standardized intervention and control conditions. The AI-personalized group showed a 10.28% GPA increase (95% CI 8.94–11.62, d = 0.89, *p* < 0.001), 36.7% stress reduction (95% CI 33.2–40.1, d = 1.42, *p* < 0.001), and 28.4% HRV improvement (95% CI 24.8–32.0, d = 1.13, *p* < 0.001). The standardized intervention group showed modest improvements (3.76% GPA increase, d = 0.34, *p* = 0.024), consistent with benefits typically observed with structured digital interventions. These between-group differences, while statistically robust, represent associations rather than confirmed causal effects; the quasi-experimental design and potential residual confounding preclude definitive causal attribution to AI personalization per se. Exploratory mediation analyses suggested that sleep quality improvement and stress reduction may partially account for academic improvements, though these post-hoc pathways require confirmation in pre-registered studies with appropriate causal inference designs.

### Limitations and interpretation caveats

Several important limitations must be considered when interpreting these findings. First, selection bias may exist due to voluntary participation and technology literacy requirements. Participants were self-selected university students who owned or were willing to use smartphones and wearables, potentially representing a more technologically engaged and motivated subset of the student population. Second, attention effects from increased monitoring and digital engagement may partially account for observed improvements. While we implemented attention-matched control conditions, residual Hawthorne effects, technology novelty, and placebo responses to AI-driven interventions cannot be fully excluded. The standardized group’s improvements suggest that approximately one-third of the observed benefits may derive from structured engagement rather than AI personalization specifically. Third, the relatively short 16-week intervention and 28-week follow-up periods may be insufficient to assess long-term sustainability, seasonal variations in academic stress, or behavioral maintenance after intervention withdrawal.

Fourth, cultural specificity substantially limits generalizability. The intervention algorithms, mindfulness content, and exercise recommendations were developed and optimized specifically for Chinese university contexts and East Asian cultural frameworks. Baseline characteristics in our sample (28–31% anxiety prevalence) differed from multinational studies reporting 35–45% prevalence^10^, suggesting cultural and contextual factors may substantially influence both baseline mental health and intervention effectiveness. Cross-cultural replication in diverse educational systems and student populations is essential before broader implementation. Fifth, the digital divide represents a critical equity concern. The system requires smartphones (iOS 12 + or Android 8+), 4G connectivity, and wearable devices, potentially excluding students from lower socioeconomic backgrounds. In our sample, 5.8% required device subsidies (¥200–300). The estimated per-student cost (¥180–280/semester, ~$25–40 USD) may be prohibitive for resource-constrained institutions. This technological requirement could inadvertently widen health disparities, as students experiencing the greatest stress may have the least access to digital interventions.

Sixth, algorithmic limitations include cold-start problems (reduced effectiveness during initial 2-week adaptation with limited individual data), potential bias against irregular schedules (18% lower adherence) and very low baseline fitness (23% higher dropout risk in bottom 10%), and “black box” interpretability challenges that may reduce user trust. Despite implementing safeguards (default safe prescriptions, weekly adherence monitoring, algorithmic fairness checks, human oversight), the system’s performance for students with comorbid conditions, disabilities, or non-traditional circumstances remains unclear. Seventh, methodological limitations include potential experimenter bias despite blinding efforts, unmeasured confounding variables, and the quasi-experimental design’s inherent inability to establish definitive causality. The large observed effect sizes (d = 0.89–1.42), while consistent with intensive multimodal interventions, warrant cautious interpretation and replication. Finally, long-term engagement challenges remain unaddressed, as digital health interventions typically show declining adherence beyond 3–6 months.

### Contributions and implications

Within these limitations, this study contributes several insights. First, it demonstrates the feasibility of implementing AI-driven personalized interventions in university settings, with 74% adherence over 16 weeks comparing favorably to typical digital intervention engagement (< 30% at 3 months). Second, the multimodal approach integrating exercise, mindfulness, and real-time biometric feedback represents an advance over single-domain interventions, with exploratory analyses suggesting potential synergistic pathways through sleep and stress regulation. Third, the hybrid deep learning framework (combining CNNs, BiLSTM networks, and reinforcement learning) offers a methodological contribution to personalized health intervention design, though further validation is needed. Fourth, the findings are consistent with a growing literature suggesting personalized interventions may enhance outcomes compared to standardized approaches^14,17^, though direct comparative effectiveness trials are needed.

For practical implementation, educational institutions should consider: (1) phased pilot deployment to assess feasibility and cultural fit; (2) integration with existing student health services for continuity of care; (3) hybrid models combining digital and face-to-face components to address equity concerns; (4) device-lending programs to ensure access for all students; and (5) approximately 8–12 h training for wellness coordinators to interpret AI-generated reports and provide support. Estimated implementation cost: $25–40 per student per semester, including subsidies and infrastructure. Implementation code, algorithm specifications, and detailed protocols are provided in Supplementary Information S1.

### Future research directions

Priority research directions include: (1) pre-registered randomized controlled trials with longer follow-up (≥ 12 months) to assess sustainability and seasonal effects; (2) cross-cultural replication in diverse educational systems (Western universities, community colleges, online programs) with attention to cultural adaptation of algorithms and content; (3) comparative effectiveness trials directly comparing AI personalization against evidence-based alternatives using equivalent resources; (4) equity-focused implementation research addressing the digital divide and evaluating hybrid delivery models; (5) mechanism studies using intensive longitudinal designs to clarify causal pathways between exercise, mindfulness, sleep, stress, and cognition; (6) algorithm improvement addressing cold-start problems, bias mitigation, and interpretability; (7) cost-effectiveness analyses for institutional decision-making; and (8) integration with clinical services to establish referral pathways for students requiring professional mental health care. Future studies should employ prospective protocol registration, active control conditions with equivalent attention, longer follow-up periods, and diverse samples to build a more robust evidence base for AI-driven student wellness interventions.

## Conclusions

This study provides preliminary evidence suggesting that AI-personalized exercise and mindfulness interventions may be associated with enhanced academic and mental health outcomes among Chinese university students compared to standardized approaches and usual care. However, substantial limitations regarding generalizability, attention effects, cultural specificity, equity concerns, and sustainability temper these findings, and the observed associations should not be interpreted as established causal effects. The results contribute to the growing field of digital mental health but require replication in diverse cultural and educational contexts with rigorous randomized methodology before broader implementation can be recommended. Educational institutions considering adoption should carefully weigh potential benefits against implementation challenges, equity concerns, and the need for complementary support services. Further research addressing the identified limitations—particularly prospective randomized trials with pre-registered hypotheses—is essential to establish the effectiveness, cost-effectiveness, and equitable implementation of AI-driven wellness interventions in higher education.

Practical implementation guidance includes: (1) phased pilot deployment to assess feasibility; (2) integration with existing student health services for referral pathways; (3) estimated cost of $25–40 per student per semester; and (4) 8–12 h training for wellness coordinators. Complete implementation code and protocols are provided in Supplementary Information S1.

Future research should prioritize: (1) longitudinal trials spanning multiple academic years to assess sustainability; (2) cross-cultural validation in diverse educational systems beyond East Asian contexts; (3) integration of additional biometric sensors for enhanced physiological monitoring; and (4) more sophisticated reinforcement learning approaches for accelerated personalization^56,57^. Priority areas include integration with campus mental health services and cost-effectiveness analysis for institutional decision-making. Critically, formally registered randomized controlled trials with prospective protocol registration would provide stronger causal evidence and support broader implementation.

The theoretical contributions of this research extend beyond educational contexts to inform precision health intervention frameworks more broadly. The documented mediating mechanisms linking physical activity, sleep quality, stress reduction, and cognitive performance contribute to theoretical models of academic achievement while providing empirical validation for integrated mind-body intervention approaches. The practical value lies in demonstrating a scalable, technology-enabled approach to student wellbeing support that can potentially address the growing mental health challenges facing university populations worldwide while simultaneously enhancing academic outcomes through evidence-based, personalized digital interventions.

## Supplementary Information

Below is the link to the electronic supplementary material.

Supplementary Material 1

## Data Availability

The datasets generated and analyzed during the current study, including group-level summary statistics, algorithm specifications, and complete analysis code, are provided in Supplementary Information S1. Raw physiological data containing personally identifiable information are not publicly available due to institutional ethics requirements (HEBU-REC-2024-031) but aggregated datasets are available from the corresponding author upon reasonable request through formal data-sharing agreements.
