# Supplementary material for: Optimization of academic performance and mental health in college students through an AI-driven personalized physical exercise and mindfulness intervention system
Source: Sci Rep. 2026 Jan 22;16:6024. doi: 10.1038/s41598-026-37028-6 (PMC12902042; doi:10.1038/s41598-026-37028-6)
Supplement: Supplementary file 1 — Supplementary Material 1 [file 41598_2026_37028_MOESM1_ESM.docx]

# Supplementary Information S1

## Tables

**Supplementary Table S1. Neural Network Architecture Specifications**

| Layer | Type | Input Dimension | Output Dimension | Activation | Parameters |
| --- | --- | --- | --- | --- | --- |
| 1 | CNN-1D | 168 × 7 | 168 × 32 | ReLU | Kernel=3, Stride=1, Padding=1 |
| 2 | MaxPool | 168 × 32 | 84 × 32 | - | Pool=2 |
| 3 | CNN-1D | 84 × 32 | 84 × 64 | ReLU | Kernel=3, Stride=1, Padding=1 |
| 4 | MaxPool | 84 × 64 | 42 × 64 | - | Pool=2 |
| 5 | BiLSTM | 42 × 64 | 42 × 128 | Tanh | Hidden=64, Bidirectional |
| 6 | Attention | 42 × 128 | 128 | Softmax | Heads=4 |
| 7 | Dense | 128 | 64 | ReLU | Dropout=0.3 |
| 8 | Output | 64 | 128 | Linear | Feature Vector |

**Supplementary Table S2. Hyperparameter Configuration**

| Parameter | Value | Search Range | Selection Method |
| --- | --- | --- | --- |
| Learning Rate | 0.001 | [0.0001, 0.01] | Grid Search |
| Batch Size | 32 | [16, 32, 64] | Grid Search |
| LSTM Hidden Units | 64 | [32, 64, 128] | Bayesian Optimization |
| Dropout Rate | 0.3 | [0.1, 0.5] | Cross-Validation |
| Attention Heads | 4 | [2, 4, 8] | Grid Search |
| Weight Decay | 1e-5 | [1e-6, 1e-4] | Grid Search |
| Epochs | 100 | - | Early Stopping (patience=10) |
| Optimizer | Adam | - | Fixed |
| Loss Function | MSE + BCE | - | Custom Combined |

**Supplementary Table S3. Mixed-Effects Model Specifications**

| Model | Fixed Effects | Random Effects | Covariance Structure |
| --- | --- | --- | --- |
| Academic Performance | Time, Group, Time×Group, Age, Gender, University, Baseline GPA | Intercept, Time (by Subject) | Unstructured |
| Psychological Health | Time, Group, Time×Group, Age, Gender, Baseline PSS | Intercept, Time (by Subject) | Unstructured |
| Physiological Parameters | Time, Group, Time×Group, Age, Gender, Baseline HRV | Intercept (by Subject) | Compound Symmetry |

**Supplementary Table S4. Data Preprocessing Parameters**

| Data Type | Preprocessing Step | Parameters |
| --- | --- | --- |
| HRV Time-Series | Bandpass Filter | 0.5-40 Hz, Butterworth 4th order |
| HRV Time-Series | Artifact Detection | ±3 SD threshold from local median |
| HRV Time-Series | Interpolation | Cubic spline for gaps <5 min |
| HRV Time-Series | RMSSD Calculation | 5-min windows, 50% overlap |
| Sleep Data | Total Sleep Time | Accelerometry threshold <0.1g |
| Sleep Data | Sleep Efficiency | TST / Time in Bed × 100% |
| Sleep Data | WASO | Minutes awake after sleep onset |
| Psychological Scores | Standardization | Z-score within timepoint |
| Academic Data | Normalization | Min-max scaling [0, 1] |

**Supplementary Table S5. Model Diagnostic Results**

| Diagnostic Test | Statistic | p-value | Interpretation |
| --- | --- | --- | --- |
| Shapiro-Wilk (Residuals) | W = 0.987 | 0.142 | Normality satisfied |
| Levene's Test | F = 1.23 | 0.294 | Homoscedasticity satisfied |
| Durbin-Watson | DW = 1.89 | 0.312 | No autocorrelation |
| VIF (max) | 2.34 | - | No multicollinearity |
| ICC (GPA) | 0.94 | - | High reliability |
| ICC (PSS) | 0.87 | - | Good reliability |
| ICC (HRV) | 0.91 | - | High reliability |

**Supplementary Table S6. Sensitivity Analysis Results**

| Analysis | Original Effect (d) | Sensitivity Effect (d) | Difference |
| --- | --- | --- | --- |
| GPA (excluding outliers ±3SD) | 0.89 | 0.82 | -0.07 |
| PSS (per-protocol only) | 1.42 | 1.38 | -0.04 |
| HRV (complete cases only) | 1.13 | 1.09 | -0.04 |
| GPA (controlling for motivation) | 0.89 | 0.76 | -0.13 |
| PSS (baseline adjustment) | 1.42 | 1.31 | -0.11 |

**Supplementary Table S7. Mediation Analysis Results (Exploratory)**

| Path | Estimate | SE | 95% CI | p-value |
| --- | --- | --- | --- | --- |
| a (Intervention → Sleep Quality) | 0.42 | 0.06 | [0.30, 0.54] | <0.001 |
| b (Sleep Quality → GPA) | 0.38 | 0.05 | [0.28, 0.48] | <0.001 |
| c' (Direct Effect) | 0.31 | 0.07 | [0.17, 0.45] | <0.001 |
| ab (Indirect Effect) | 0.16 | 0.03 | [0.10, 0.22] | <0.001 |
| a (Intervention → Stress Reduction) | 0.51 | 0.05 | [0.41, 0.61] | <0.001 |
| b (Stress Reduction → GPA) | 0.35 | 0.06 | [0.23, 0.47] | <0.001 |
| ab (Indirect Effect) | 0.18 | 0.04 | [0.12, 0.25] | <0.001 |

## Code

**S1.1 Data Preprocessing Pipeline (Python)**

import numpy as np
import pandas as pd
from scipy.signal import butter, filtfilt
from scipy.interpolate import CubicSpline

class HRVPreprocessor:
 def __init__(self, fs=100, lowcut=0.5, highcut=40, order=4):
 self.fs = fs
 self.lowcut = lowcut
 self.highcut = highcut
 self.order = order

 def bandpass_filter(self, data):
 nyq = 0.5 * self.fs
 low = self.lowcut / nyq
 high = self.highcut / nyq
 b, a = butter(self.order, [low, high], btype='band')
 return filtfilt(b, a, data)

 def detect_artifacts(self, ibi_series, threshold=3):
 local_median = ibi_series.rolling(window=10, center=True).median()
 deviation = np.abs(ibi_series - local_median) / local_median
 artifacts = deviation > (threshold * deviation.std())
 return artifacts

 def interpolate_gaps(self, data, timestamps, max_gap_minutes=5):
 max_gap_samples = max_gap_minutes * 60 * self.fs
 valid_idx = ~np.isnan(data)
 if valid_idx.sum() < 2:
 return data
 cs = CubicSpline(timestamps[valid_idx], data[valid_idx])
 interpolated = cs(timestamps)
 gap_lengths = np.diff(np.where(valid_idx)[0])
 for i, gap in enumerate(gap_lengths):
 if gap > max_gap_samples:
 start_idx = np.where(valid_idx)[0][i]
 end_idx = np.where(valid_idx)[0][i+1]
 interpolated[start_idx:end_idx] = np.nan
 return interpolated

 def calculate_rmssd(self, ibi_series, window_sec=300, overlap=0.5):
 window_size = int(window_sec * self.fs)
 step_size = int(window_size * (1 - overlap))
 rmssd_values = []
 for start in range(0, len(ibi_series) - window_size, step_size):
 window = ibi_series[start:start + window_size]
 if np.isnan(window).sum() / len(window) < 0.1:
 diff = np.diff(window)
 rmssd = np.sqrt(np.nanmean(diff ** 2))
 rmssd_values.append(rmssd)
 else:
 rmssd_values.append(np.nan)
 return np.array(rmssd_values)

 def process(self, raw_data, timestamps):
 filtered = self.bandpass_filter(raw_data)
 artifacts = self.detect_artifacts(pd.Series(filtered))
 filtered[artifacts] = np.nan
 interpolated = self.interpolate_gaps(filtered, timestamps)
 rmssd = self.calculate_rmssd(interpolated)
 return {'filtered': interpolated, 'rmssd': rmssd}

**S1.2 Student Feature Analysis Model (PyTorch)**

import torch
import torch.nn as nn

class StudentFeatureModel(nn.Module):
 def __init__(self, input_channels=7, seq_length=168, hidden_dim=64,
 output_dim=128, num_heads=4, dropout=0.3):
 super(StudentFeatureModel, self).__init__()

 # CNN layers for feature extraction
 self.conv1 = nn.Conv1d(input_channels, 32, kernel_size=3, padding=1)
 self.conv2 = nn.Conv1d(32, 64, kernel_size=3, padding=1)
 self.pool = nn.MaxPool1d(2)
 self.bn1 = nn.BatchNorm1d(32)
 self.bn2 = nn.BatchNorm1d(64)

 # BiLSTM for temporal processing
 self.lstm = nn.LSTM(64, hidden_dim, num_layers=2,
 batch_first=True, bidirectional=True,
 dropout=dropout)

 # Multi-head attention
 self.attention = nn.MultiheadAttention(hidden_dim * 2, num_heads,
 dropout=dropout)
 self.attention_norm = nn.LayerNorm(hidden_dim * 2)

 # Output layers
 self.fc1 = nn.Linear(hidden_dim * 2, hidden_dim)
 self.fc2 = nn.Linear(hidden_dim, output_dim)
 self.dropout = nn.Dropout(dropout)
 self.relu = nn.ReLU()

 def forward(self, x):
 # x shape: (batch, channels, seq_length)
 x = self.relu(self.bn1(self.conv1(x)))
 x = self.pool(x)
 x = self.relu(self.bn2(self.conv2(x)))
 x = self.pool(x)

 # Reshape for LSTM: (batch, seq, features)
 x = x.permute(0, 2, 1)
 lstm_out, _ = self.lstm(x)

 # Attention mechanism
 attn_in = lstm_out.permute(1, 0, 2)
 attn_out, attn_weights = self.attention(attn_in, attn_in, attn_in)
 attn_out = self.attention_norm(attn_out + attn_in)

 # Global pooling
 pooled = attn_out.mean(dim=0)

 # Output projection
 out = self.dropout(self.relu(self.fc1(pooled)))
 out = self.fc2(out)

 return out, attn_weights

class CustomLoss(nn.Module):
 def __init__(self, alpha=0.5, beta=0.3, gamma=0.2):
 super(CustomLoss, self).__init__()
 self.alpha = alpha # prediction accuracy weight
 self.beta = beta # engagement weight
 self.gamma = gamma # safety weight
 self.mse = nn.MSELoss()
 self.bce = nn.BCEWithLogitsLoss()

 def forward(self, pred, target, engagement_pred, engagement_target,
 safety_flags):
 pred_loss = self.mse(pred, target)
 engage_loss = self.bce(engagement_pred, engagement_target)
 safety_loss = (safety_flags * torch.abs(pred - target)).mean()
 total = self.alpha * pred_loss + self.beta * engage_loss + \
 self.gamma * safety_loss
 return total

**S1.3 Exercise Matching Model (FM-DNN)**

import torch
import torch.nn as nn

class FactorizationMachine(nn.Module):
 def __init__(self, n_features, n_factors=10):
 super(FactorizationMachine, self).__init__()
 self.linear = nn.Linear(n_features, 1)
 self.v = nn.Parameter(torch.randn(n_features, n_factors) * 0.01)

 def forward(self, x):
 linear_part = self.linear(x)
 square_of_sum = torch.pow(torch.matmul(x, self.v), 2)
 sum_of_square = torch.matmul(torch.pow(x, 2), torch.pow(self.v, 2))
 interaction = 0.5 * torch.sum(square_of_sum - sum_of_square,
 dim=1, keepdim=True)
 return linear_part + interaction

class ExerciseMatchingModel(nn.Module):
 def __init__(self, student_dim=128, exercise_dim=64, context_dim=32,
 hidden_dims=[256, 128, 64], n_factors=10, dropout=0.3):
 super(ExerciseMatchingModel, self).__init__()

 total_input = student_dim + exercise_dim + context_dim

 # Factorization Machine component
 self.fm = FactorizationMachine(total_input, n_factors)

 # Deep Neural Network component
 layers = []
 prev_dim = total_input
 for hidden_dim in hidden_dims:
 layers.extend([
 nn.Linear(prev_dim, hidden_dim),
 nn.ReLU(),
 nn.BatchNorm1d(hidden_dim),
 nn.Dropout(dropout)
 ])
 prev_dim = hidden_dim
 self.dnn = nn.Sequential(*layers)
 self.output = nn.Linear(hidden_dims[-1] + 1, 1)
 self.sigmoid = nn.Sigmoid()

 def forward(self, student_features, exercise_features, context_features):
 x = torch.cat([student_features, exercise_features,
 context_features], dim=1)
 fm_out = self.fm(x)
 dnn_out = self.dnn(x)
 combined = torch.cat([fm_out, dnn_out], dim=1)
 out = self.sigmoid(self.output(combined))
 return out

**S1.4 Mindfulness Adaptation Model (GCN)**

import torch
import torch.nn as nn
import torch.nn.functional as F

class GraphConvLayer(nn.Module):
 def __init__(self, in_features, out_features):
 super(GraphConvLayer, self).__init__()
 self.weight = nn.Parameter(torch.FloatTensor(in_features, out_features))
 self.bias = nn.Parameter(torch.FloatTensor(out_features))
 nn.init.xavier_uniform_(self.weight)
 nn.init.zeros_(self.bias)

 def forward(self, x, adj):
 support = torch.matmul(x, self.weight)
 output = torch.matmul(adj, support) + self.bias
 return output

class MindfulnessGCN(nn.Module):
 def __init__(self, n_psych_states=20, n_techniques=15, n_contexts=10,
 embedding_dim=64, hidden_dim=128, output_dim=64,
 n_layers=3, dropout=0.3):
 super(MindfulnessGCN, self).__init__()

 # Node embeddings
 self.psych_embed = nn.Embedding(n_psych_states, embedding_dim)
 self.tech_embed = nn.Embedding(n_techniques, embedding_dim)
 self.context_embed = nn.Embedding(n_contexts, embedding_dim)

 # Graph convolution layers
 self.gc_layers = nn.ModuleList()
 self.gc_layers.append(GraphConvLayer(embedding_dim, hidden_dim))
 for _ in range(n_layers - 2):
 self.gc_layers.append(GraphConvLayer(hidden_dim, hidden_dim))
 self.gc_layers.append(GraphConvLayer(hidden_dim, output_dim))

 # Output prediction
 self.predictor = nn.Sequential(
 nn.Linear(output_dim * 2, hidden_dim),
 nn.ReLU(),
 nn.Dropout(dropout),
 nn.Linear(hidden_dim, 1),
 nn.Sigmoid()
 )
 self.dropout = nn.Dropout(dropout)

 def forward(self, psych_ids, tech_ids, context_ids, adj_matrix):
 # Get embeddings
 psych_emb = self.psych_embed(psych_ids)
 tech_emb = self.tech_embed(tech_ids)
 context_emb = self.context_embed(context_ids)

 # Concatenate all node features
 x = torch.cat([psych_emb, tech_emb, context_emb], dim=0)

 # Graph convolutions
 for i, gc in enumerate(self.gc_layers[:-1]):
 x = F.relu(gc(x, adj_matrix))
 x = self.dropout(x)
 x = self.gc_layers[-1](x, adj_matrix)

 # Extract relevant node representations
 n_psych = psych_ids.size(0)
 n_tech = tech_ids.size(0)
 psych_repr = x[:n_psych].mean(dim=0)
 tech_repr = x[n_psych:n_psych + n_tech]

 # Predict effectiveness for each technique
 combined = torch.cat([psych_repr.unsqueeze(0).expand(n_tech, -1),
 tech_repr], dim=1)
 effectiveness = self.predictor(combined)

 return effectiveness

**S1.5 Multi-objective Optimization**

import numpy as np
from scipy.optimize import minimize

class MultiObjectiveOptimizer:
 def __init__(self, weights={'academic': 0.35, 'stress': 0.30,
 'physical': 0.20, 'engagement': 0.15}):
 self.weights = weights

 def objective_function(self, x, student_profile, models):
 academic_pred = models['academic'].predict(x, student_profile)
 stress_pred = models['stress'].predict(x, student_profile)
 physical_pred = models['physical'].predict(x, student_profile)
 engagement_pred = models['engagement'].predict(x, student_profile)

 total = -(self.weights['academic'] * academic_pred +
 self.weights['stress'] * (1 - stress_pred) +
 self.weights['physical'] * physical_pred +
 self.weights['engagement'] * engagement_pred)
 return total

 def safety_constraints(self, x, student_profile):
 constraints = []
 max_hr = 220 - student_profile['age']
 constraints.append(x[0] - 0.85 * max_hr) # HR <= 85% max
 constraints.append(x[1] - 90) # Duration <= 90 min
 constraints.append(3 - x[2]) # Rest days >= 3 per week
 return np.array(constraints)

 def optimize(self, student_profile, models, bounds):
 x0 = np.array([0.6 * (220 - student_profile['age']), 30, 4])

 constraints = {'type': 'ineq',
 'fun': lambda x: -self.safety_constraints(x, student_profile)}

 result = minimize(
 self.objective_function,
 x0,
 args=(student_profile, models),
 method='SLSQP',
 bounds=bounds,
 constraints=constraints,
 options={'maxiter': 100}
 )

 return {
 'target_hr': result.x[0],
 'duration_minutes': result.x[1],
 'sessions_per_week': int(result.x[2]),
 'optimization_success': result.success
 }

**S1.6 Mixed-Effects Model Analysis (R)**

# Load required packages
library(lme4)
library(lmerTest)
library(emmeans)
library(performance)

# Model specification for academic performance
fit_academic <- lmer(
 GPA ~ Time * Group + Age + Gender + University + Baseline_GPA +
 (1 + Time | SubjectID),
 data = academic_data,
 REML = TRUE,
 control = lmerControl(optimizer = "bobyqa", optCtrl = list(maxfun = 100000))
)

# Model diagnostics
check_model(fit_academic)
check_normality(fit_academic)
check_heteroscedasticity(fit_academic)

# Extract fixed effects with confidence intervals
summary(fit_academic)
confint(fit_academic, method = "Wald")

# Estimated marginal means
emm_results <- emmeans(fit_academic, ~ Group | Time)
pairs(emm_results, adjust = "tukey")

# Effect size calculation
effect_sizes <- function(model, group_var = "Group") {
 fixef_est <- fixef(model)
 sigma <- sigma(model)
 d <- fixef_est[grep(group_var, names(fixef_est))] / sigma
 return(d)
}

# Model comparison for random effects structure
fit_null <- lmer(GPA ~ Time * Group + Age + Gender + (1 | SubjectID),
 data = academic_data, REML = FALSE)
fit_full <- lmer(GPA ~ Time * Group + Age + Gender + (1 + Time | SubjectID),
 data = academic_data, REML = FALSE)
anova(fit_null, fit_full)

# Benjamini-Hochberg correction
p_values <- c(p_gpa, p_pss, p_hrv, p_exam, p_cognitive)
p_adjusted <- p.adjust(p_values, method = "BH")

**S1.7 Structural Equation Modeling for Mediation (R)**

# Load required package
library(lavaan)

# Define mediation model
mediation_model <- '
 # Measurement model (if applicable)

 # Direct effects
 GPA_change ~ c*Group

 # Mediator paths
 Sleep_quality ~ a1*Group
 Stress_reduction ~ a2*Group

 GPA_change ~ b1*Sleep_quality
 GPA_change ~ b2*Stress_reduction

 # Indirect effects
 indirect1 := a1*b1
 indirect2 := a2*b2
 total_indirect := indirect1 + indirect2
 total := c + total_indirect

 # Covariances
 Sleep_quality ~~ Stress_reduction
'

# Fit model with bootstrap for confidence intervals
fit_mediation <- sem(
 mediation_model,
 data = mediation_data,
 se = "bootstrap",
 bootstrap = 5000,
 estimator = "ML"
)

# Summary with standardized estimates
summary(fit_mediation, standardized = TRUE, fit.measures = TRUE, ci = TRUE)

# Extract specific indirect effects
parameterEstimates(fit_mediation, boot.ci.type = "bca.simple",
 level = 0.95, ci = TRUE)

# Model fit indices
fitMeasures(fit_mediation, c("cfi", "tli", "rmsea", "srmr"))

**S1.8 SHAP Feature Importance Analysis (Python)**

import shap
import numpy as np
from sklearn.ensemble import RandomForestClassifier
from sklearn.model_selection import cross_val_score

class SHAPAnalyzer:
 def __init__(self, model, X_train, feature_names):
 self.model = model
 self.X_train = X_train
 self.feature_names = feature_names
 self.explainer = shap.TreeExplainer(model)

 def compute_shap_values(self, X_test):
 shap_values = self.explainer.shap_values(X_test)
 return shap_values

 def get_feature_importance(self, shap_values):
 if isinstance(shap_values, list):
 shap_values = shap_values[1]
 importance = np.abs(shap_values).mean(axis=0)
 importance_normalized = importance / importance.max() * 100
 feature_importance = dict(zip(self.feature_names, importance_normalized))
 return dict(sorted(feature_importance.items(),
 key=lambda x: x[1], reverse=True))

 def plot_summary(self, shap_values, X_test, save_path=None):
 shap.summary_plot(shap_values, X_test,
 feature_names=self.feature_names,
 show=False)
 if save_path:
 import matplotlib.pyplot as plt
 plt.savefig(save_path, dpi=300, bbox_inches='tight')
 plt.close()

# Usage example
feature_names = [
 'sleep_quality_consistency', 'stress_reactivity', 'exercise_adherence',
 'baseline_hrv', 'academic_motivation', 'mindfulness_frequency',
 'chronotype_alignment', 'social_support', 'baseline_fitness',
 'learning_style_match'
]

# Train responder classification model
rf_model = RandomForestClassifier(
 n_estimators=100,
 max_depth=10,
 random_state=42,
 class_weight='balanced'
)
rf_model.fit(X_train, y_train)

# Cross-validation accuracy
cv_scores = cross_val_score(rf_model, X_train, y_train, cv=10)
print(f"10-fold CV Accuracy: {cv_scores.mean():.3f} (+/- {cv_scores.std()*2:.3f})")

# SHAP analysis
analyzer = SHAPAnalyzer(rf_model, X_train, feature_names)
shap_values = analyzer.compute_shap_values(X_test)
importance = analyzer.get_feature_importance(shap_values)

for feature, imp in importance.items():
 print(f"{feature}: {imp:.1f}%")

**S1.9 Online Learning Update Algorithm**

import torch
import torch.optim as optim
from collections import deque

class OnlineLearner:
 def __init__(self, model, base_lr=0.001, memory_size=1000):
 self.model = model
 self.base_lr = base_lr
 self.memory = deque(maxlen=memory_size)
 self.optimizer = optim.Adam(model.parameters(), lr=base_lr)
 self.scheduler = optim.lr_scheduler.ReduceLROnPlateau(
 self.optimizer, mode='min', factor=0.5, patience=5
 )

 def adaptive_learning_rate(self, hrv_trend, stress_level):
 """Increase sensitivity during detected stress periods"""
 if hrv_trend < -0.1 or stress_level > 0.7:
 lr_multiplier = 1.5
 elif hrv_trend > 0.1 and stress_level < 0.3:
 lr_multiplier = 0.8
 else:
 lr_multiplier = 1.0

 for param_group in self.optimizer.param_groups:
 param_group['lr'] = self.base_lr * lr_multiplier

 def update(self, new_data, outcome):
 self.memory.append((new_data, outcome))

 # Sample mini-batch from memory
 if len(self.memory) >= 32:
 batch_idx = torch.randint(0, len(self.memory), (32,))
 batch_data = [self.memory[i][0] for i in batch_idx]
 batch_outcomes = [self.memory[i][1] for i in batch_idx]

 # Forward pass
 self.model.train()
 predictions = self.model(torch.stack(batch_data))
 loss = self.compute_loss(predictions, torch.tensor(batch_outcomes))

 # Backward pass
 self.optimizer.zero_grad()
 loss.backward()
 torch.nn.utils.clip_grad_norm_(self.model.parameters(), max_norm=1.0)
 self.optimizer.step()

 self.scheduler.step(loss)

 return loss.item()
 return None

 def compute_loss(self, predictions, targets):
 mse_loss = torch.nn.functional.mse_loss(predictions.squeeze(),
 targets.float())
 return mse_loss

**S1.10 Cold-Start Recommendation Strategy**

import numpy as np
from sklearn.neighbors import NearestNeighbors

class ColdStartHandler:
 def __init__(self, historical_data, n_neighbors=5):
 self.historical_data = historical_data
 self.demographic_features = ['age', 'gender', 'fitness_level',
 'academic_year', 'major_category']
 self.knn = NearestNeighbors(n_neighbors=n_neighbors, metric='euclidean')
 self._fit_knn()

 def _fit_knn(self):
 X = self.historical_data[self.demographic_features].values
 X_normalized = (X - X.mean(axis=0)) / X.std(axis=0)
 self.knn.fit(X_normalized)
 self.X_normalized = X_normalized

 def get_initial_recommendation(self, new_user_profile):
 user_features = np.array([[new_user_profile[f]
 for f in self.demographic_features]])
 user_normalized = (user_features - self.historical_data[
 self.demographic_features].mean().values) / \
 self.historical_data[self.demographic_features].std().values

 distances, indices = self.knn.kneighbors(user_normalized)
 similar_users = self.historical_data.iloc[indices[0]]

 # Weighted average of successful interventions
 weights = 1 / (distances[0] + 1e-6)
 weights = weights / weights.sum()

 recommendation = {
 'exercise_type': similar_users['best_exercise_type'].mode()[0],
 'exercise_intensity': np.average(
 similar_users['optimal_intensity'], weights=weights),
 'exercise_duration': np.average(
 similar_users['optimal_duration'], weights=weights),
 'mindfulness_technique': similar_users['best_mindfulness'].mode()[0],
 'mindfulness_duration': np.average(
 similar_users['optimal_mind_duration'], weights=weights),
 'confidence': 1 - distances[0].mean()
 }

 # Apply conservative safety bounds
 recommendation['exercise_intensity'] = min(
 recommendation['exercise_intensity'], 0.65)
 recommendation['exercise_duration'] = min(
 recommendation['exercise_duration'], 30)

 return recommendation

 def get_default_safe_prescription(self, stress_level='moderate'):
 """Default prescriptions when no similar users found"""
 defaults = {
 'low': {
 'exercise_type': 'moderate_aerobic',
 'exercise_intensity': 0.55,
 'exercise_duration': 25,
 'mindfulness_technique': 'body_scan',
 'mindfulness_duration': 10
 },
 'moderate': {
 'exercise_type': 'light_aerobic',
 'exercise_intensity': 0.50,
 'exercise_duration': 20,
 'mindfulness_technique': 'breathing_focus',
 'mindfulness_duration': 10
 },
 'high': {
 'exercise_type': 'gentle_stretching',
 'exercise_intensity': 0.40,
 'exercise_duration': 15,
 'mindfulness_technique': 'guided_relaxation',
 'mindfulness_duration': 15
 }
 }
 return defaults.get(stress_level, defaults['moderate'])
